# Synthetic promoters developed through a site-specific integration system enhance antibody expression in fed-batch culture

**DOI:** 10.48130/els-0026-0004

**Published:** 2026-07-24

**Authors:** Liyun Yue, Hao Li, Songtao Zhou

**Affiliations:** Department of New Technology Development, Shanghai Hengrui Pharmaceutical Co., Ltd, Shanghai 200245, China

**Keywords:** Landing pad locus-specific, Antibody expression, Synthetic promoters, Site-specific integration, Chinese hamster ovary (CHO) cells

## Abstract

Constructing expression cell lines using site-specific integration (SSI) technology has become a prominent focus in cell line development (CLD) because of its potential to minimize clonal variation. However, this technology has not yet been widely adopted in commercial biopharmaceutical manufacturing. A major reason is the low yield of SSI-derived cell lines. To improve yield, we designed landing pad locus-specific synthetic promoters by screening for potent transcription factor regulatory elements (TFREs) within an SSI platform. We then combined these TFREs to create novel, strong promoters. All synthetic promoters exhibited reporter expression comparable to that of the CMV promoter, with some up to 20% stronger, despite being only one-third the length of the CMV promoter. When expressing a monospecific antibody, fed-batch titers from these synthetic promoters were up to 45% higher than those from the CMV promoter. We also used synthetic promoters to express a bispecific antibody via SSI. The titer from a pure synthetic promoter combination was ~2.7 g/L, lower than that from an all-CMV promoter combination (~3.8 g/L). These findings demonstrate that established SSI platforms can be used to design efficient context-specific promoters, leading to significant improvements in antibody expression and greatly enhancing the value of SSI applications in CLD.

## Introduction

Monoclonal antibody (mAb)-based products are the most lucrative single product class among biopharmaceuticals. Their total sales reached ${\$} $217 billion in 2021, accounting for 15 of the top 20 products by sales. From January 2018 to June 2022, 197 biopharmaceutical products were approved, of which mAbs remained dominant, representing 53.5% of all approvals. Furthermore, there are currently more than 100 mAb-based products in late-stage clinical development^[1]^. As more candidate molecules enter the development pipeline, there is an increasing need to accelerate the cell line development process.

In the conventional Chinese hamster ovary (CHO) cell line development process, the linearized glutamine synthetase (GS) vector integrates at naturally occurring double-strand breaks in the genome, which are thought to be repaired by non-homologous end-joining, in a process known as random integration (RI). This process is unpredictable, and both the number and orientation of integrated gene copies, as well as the expression characteristics of the integration sites, can vary substantially. Consequently, labor-intensive screening of thousands of cell clones is necessary to identify those with high productivity and stability^[2,3]^. Several strategies have been developed to optimize vector sequences, enabling scientists to achieve more stable and higher expression levels in cells with reduced labor. These strategies include the use of scaffold/matrix attachment region (S/MAR)^[4]^ and ubiquitous chromatin opening elements (UCOE)^[5]^. Site-specific integration (SSI) allows for the recombination of plasmid DNA into a pre-determined genomic locus, thereby reducing clonal variation and accelerating the cell line development process^[6]^. Several methods for targeted integration of exogenous sequences have been developed, using site-specific recombinases such as Cre and Flp for recombinase-mediated cassette exchange (RMCE), as well as programmable nucleases such as CRISPR/Cas9 (clustered regularly interspaced short palindromic repeat/CRISPR-associated protein 9)^[7,8]^.

Although the SSI platform has been reported and tested in some pharmaceutical companies^[3,9]^, its applications in final commercial manufacturing remain limited, primarily because of the low yield associated with single-copy gene inserts. One possible solution is to increase the copy number of the inserted genes. Carver et al. reported that their mAb titers increased to 2–3 g/L after the addition of two or three copies of both the light chain (LC) and heavy chain (HC) genes, whereas the original titer was only 1 g/L with a single copy of both LC and HC genes^[9]^. Although this method is straightforward for increasing titers, constructing target gene concatemers can be challenging^[2]^.

Promoter optimization offers a promising approach to improve yield. Currently, viral sequence-based promoters, such as the cytomegalovirus (CMV) promoter, and endogenous promoters like the EF1*α* promoter, dominate cell line development. To maintain high expression levels, these naturally derived promoters are typically lengthy, which may adversely affect the integration efficiency of RMCE^[2,10,11]^. Synthetic promoters are custom-designed to eliminate functionally ill-defined and uncontrollable elements^[11,12]^ in expression vectors. This design yields shorter synthetic promoters, which are expected to improve RMCE efficiency. In addition, Brown et al.^[12]^ reported that their newly developed synthetic promoters were already stronger than the commonly used hCMV-IE1 promoter, further demonstrating the potential of synthetic promoters. Furthermore, diverse synthetic promoters and their combinations can provide precise control over recombinant transcriptional activity in cells^[13]^, which is a significant advantage for enhancing the product quality of complex molecules, such as bispecific antibodies (bsAbs)^[14]^. However, current synthetic promoter designs do not take the locus effect into account.

In this study, we are the first to describe the construction of several efficient landing pad locus-specific synthetic promoters using the SSI platform. This was achieved by screening multiple transcription factor regulatory elements (TFREs, or transcription factor binding sites) and identifying the optimal copy number for each TFRE. When using SSI to express a reporter gene, these landing pad locus-specific synthetic promoters demonstrated greater strength than the previously reported synthetic promoter 100RPU.2^[12]^, which was designed based on a transient expression platform. The synthetic promoters, comprising ~30 TFREs, were further employed to express monospecific antibodies (msAbs) in CHO-K1 cells. We observed that both the titer and targeted integration efficiency were improved when using synthetic promoters compared to the proprietary CMV promoter^[15]^. Finally, when the synthetic promoter was used to express bsAbs in a four-in-one vector system, the titer level was lower than that of the CMV promoter, indicating that the current system needs improvement to accommodate the expression of complex molecules.

## Results

### Establishment of an SSI-based synthetic promoter screening platform

**Figure 1:**
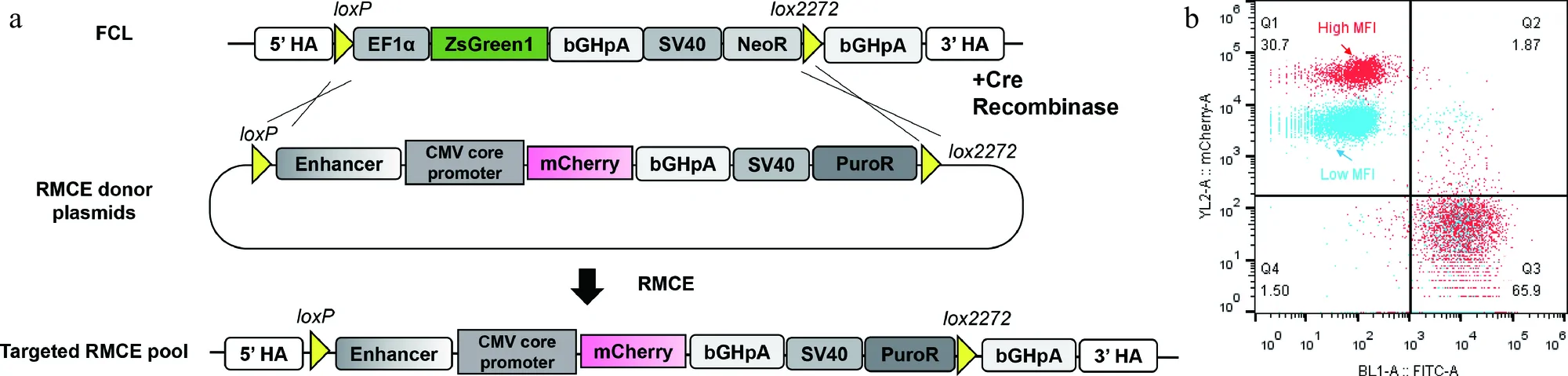
Generation of a synthetic promoter screening platform via SSI and its application in promoter screening. (a) We previously integrated the landing pad (LP) sequence into a safe harbor site within the Chinese hamster ovary (CHO-K1) cell genome to construct a founder cell line (FCL). Within the LP, the ZsGreen1 expression cassette is flanked by *loxP* and *lox2272* sites, with a bGH poly(A) sequence located downstream of the *lox2272* site. Upon co-transfection with the Cre recombinase plasmid, the ZsGreen1 expression cassette is replaced by the mCherry expression cassette from the RMCE donor plasmid. The correctly integrated cells (mCherry+/ZsGreen1-) are enriched under puromycin selection for 10 d. By measuring the median fluorescence intensity (MFI) of the successfully recombined populations, the expression levels of different synthetic promoters were evaluated. HA: homologous arm. (b) By detecting the MFI of the successfully recombined populations (i.e., mCherry+/ZsGReen1- cells in quadrant Q1), we can distinguish the strength of the two different promoters. Quadrant Q1 merged the flow cytometry results of the two promoters with varying strengths. The cell population in quadrant Q3 was resistant to puromycin selection and did not undergo recombination at the target locus.

Promoters are among the most fundamental components of synthetic biological systems. The performance of a promoter is generally context-dependent, meaning that transcription levels are modulated by the surrounding genetic sequence^[16]^. To more accurately assess the strength of synthetic promoters, we developed a poly(A) trap-based RMCE landing pad, enabling systematic evaluation of recombinant gene expression levels in our SSI platform (Fig. 1a). The founder cell line (FCL) was previously established by engineering a CHO-K1-derived cell line with landing pads flanked by distinct *lox* sites through homologous-dependent recombination (HDR). The FCL design incorporates an EF1*α* promoter-ZsGreen1-bGH poly(A)-SV40 promoter-NeoR inside the landing pad and bGH poly(A) outside the landing pad to facilitate poly(A) trapping. We also constructed an RMCE donor plasmid, comprising a synthetic promoter (consisting of an enhancer and CMV core promoter) followed by the mCherry gene (with poly[A]), alongside a puromycin resistance selection cassette (PuroR) lacking poly(A). This donor plasmid is capable of undergoing RMCE to replace the sequence within the landing pad of the FCL with the poly(A) trap. Once the donor sequence is correctly integrated, the recombinant cells can survive puromycin selection. This approach ensures efficient insertion of desired expression cassettes into the same landing pad locus, allowing for the measurement of expression differences driven by synthetic promoter variants (Fig. 1a). Exemplar mCherry^+^/ZsGreen1^−^ populations were observed, demonstrating successful recombination and tunable mCherry transcriptional output from the poly(A) trap (Fig. 1b), thereby validating the effectiveness of our screening platform.

To verify this workflow, we constructed five different mCherry constructs with various enhancer parts to replace the ZsGreen1 expression cassette. Following puromycin selection, mCherry^+^/ZsGreen1^−^ populations were enriched and detected, demonstrating successful recombination and transcriptional output of mCherry from the poly(A) trap. The median fluorescence intensities (MFIs) of mCherry in the recombined cells showed significant variation across the different promoters (Supplementary Fig. S1a). Notably, the proportion of integrated cells (calculated as Q1/[Q1 + Q3]) associated with the CMV promoter^[15]^ was substantially lower (~1.7%) compared to other promoters, which ranged from approximately 30% to 80% (Promoters 1–3). This observation suggests that shorter DNA sequences are exchanged more efficiently, given that Promoters 1–3 are approximately 200 bp, whereas the CMV promoter spans roughly 1,500 bp. Subsequently, we performed bulk sorting of the mCherry^+^/ZsGreen1^−^ populations for all constructs. The sorted cells were expanded and lysed for genomic DNA extraction. Both 5' and 3' junction PCR results (Supplementary Fig. S2) of the bulk-sorted samples confirmed the correct integration of the constructs (Supplementary Fig. S1b). Furthermore, all bulk-sorted populations exhibited a 1:1 target-to-reference ratio, indicating that, on average, these populations contained a single copy of the mCherry gene (Supplementary Table S1).

### Homotypic clusters of TFREs exhibit varied reporter expression levels

Several studies have reported using transient transfection to assess the transgenic expression levels of homotypic promoters in CHO cells^[11,12]^. In the present study, we selected a total of 44 distinct TFREs (Supplementary Table S2) from both the literature and a web-based TFRE prediction tool (TRAP [mpg.de]) to identify potential TFRE candidates from the proprietary CMV promoter^[15]^. These TFREs were concatenated with eight copies to construct homotypic functional sequences, with each TFRE separated by a 3-bp spacer. The measurement of MFI derived from SSI of each reporter expression cassette is presented in Supplementary Fig. S3a. In the SSI platform, five TFRE types—REL, NF*κ*B, AP1, NF*κ*B-p65, and ERSE—were identified as the most potent, exhibiting expression levels exceeding those of the minimal core promoter by more than two orders of magnitude. In addition, 16 other TFREs (DRE, RELA, GABP*β*, USF1, ARE, Sp1, AhR/ARNT, MZF1, E-box, GC-box, DMP1, D-box, EBS1, NF1-RE, AARE, and Pax3) showed expression exceeding the control by more than one order of magnitude. Despite the relatively short length of these synthetic promoters—comprising eight TFREs and approximately 200 bp, which was roughly one-seventh the length of the CMV promoter—they accounted for 45% of the CMV promoter's expression level. Furthermore, we tested a previously reported synthetic promoter^[12]^, 100RPU.2, in the SSI platform. This promoter achieved 70% of the CMV promoter's strength despite being only 25% in length. All these results demonstrated the potential of synthetic promoters in SSI applications. Additionally, mCherry expression levels were recorded 3 d after transient transfection (Supplementary Fig. S3b). The distribution of reporter expression was broad in transient transfection, probably due to the variations of transfection efficiency among individual cells. In contrast, the distribution of reporter expression in the SSI platform was more centralized, indicating a high degree of consistency in assessing TFRE strength via SSI. Interestingly, many TFREs exhibited similar ranking patterns across both methods (Supplementary Fig. S3a, S3b), with some exceptions observed, such as RELA vs NF*κ*B, ERSE vs RELA, AP1 vs NF*κ*B-p65, and RELA vs AP1. Additionally, some homotypic TFREs, including USF1 and ARE, showed minimal reporter expression in transient transfection, but demonstrated medium-level expression in the SSI platform (Supplementary Fig. S3a, S3b).

**Figure 2:**
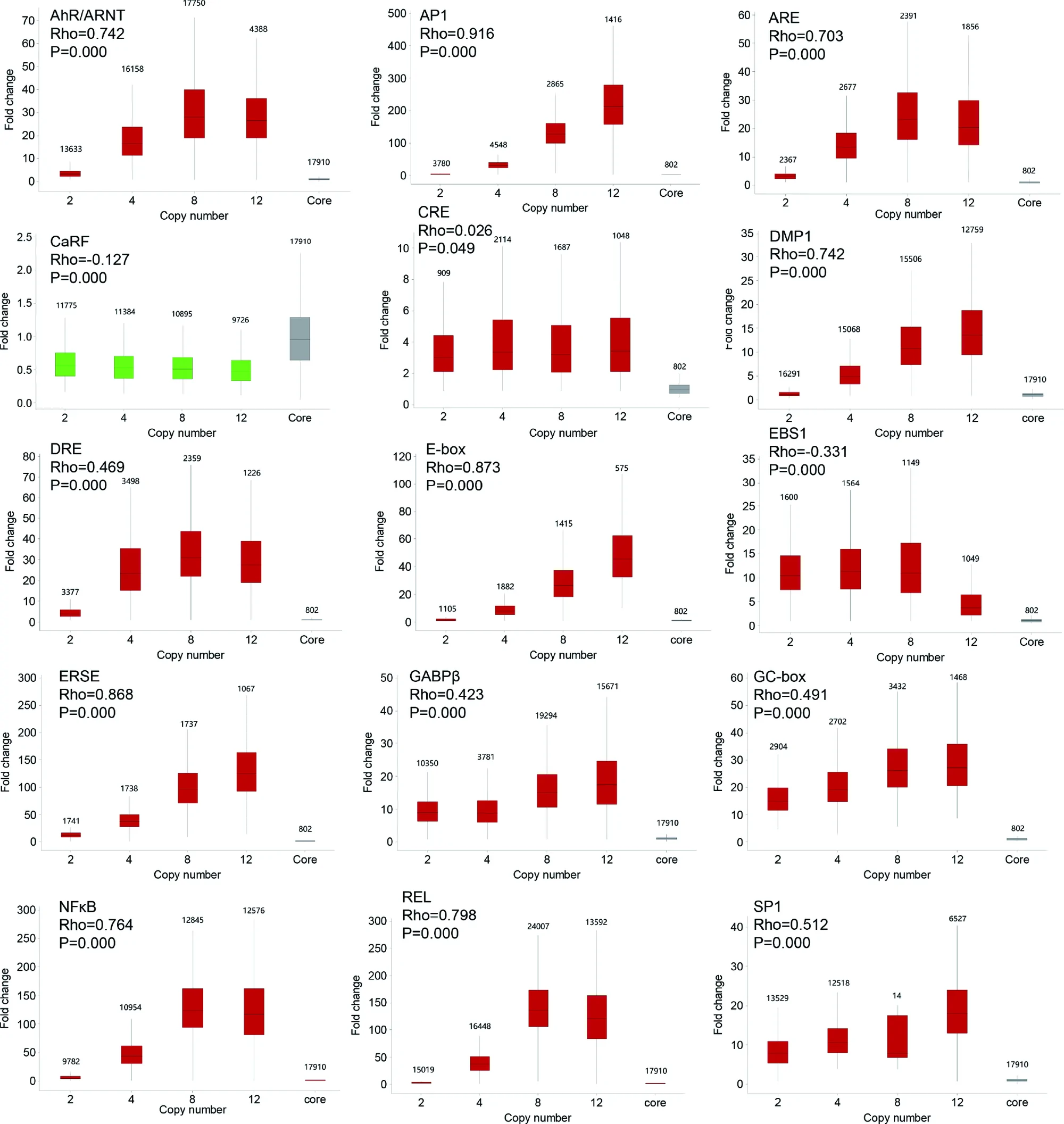
Determination of optimal copy number for 15 TFREs. Spearman's rho was used to calculate the correlations between copy number and expression level for all 15 TFREs. Benjamini–Hochberg FDR-adjusted *p*-values were derived from Spearman's correlations. The CaRF binding site was included as an example of a site that could not be homotypically amplified. On the right, box plots illustrate the background expression of the CMV core promoter without any TFREs upstream. Red boxes indicate groups of TFREs with significantly higher expression compared to the core background (Mann–Whitney U test, *p* < 0.05), while green boxes represent groups with significantly lower expression. In the box plots, the central rectangle spans the first and third quartiles, the line within the rectangle denotes the median, and the ends of the vertical lines extending beyond the box indicate the minimum and maximum values. The numbers at the top of the boxes represent the number of tested biological replicates.

A prior study has reported that the homotypic amplification of TFRE may be susceptible to saturation^[17]^. Consequently, it is essential to identify the optimal copy number for each TFRE. We selected 15 distinct TFRE types with varying expression levels—namely AhR/ARNT, AP1, ARE, CaRF, CRE, DMP1, DRE, E-box, EBS1, ERSE, GABP*β*, GC-box, NF*κ*B, REL, and SP1—and constructed homotypic TFRE enhancers by concatenating these elements in configurations of two, four, eight, and twelve copies. For eight TFREs (AhR/ARNT, AP1, ARE, DMP1, E-box, ERSE, NF*κ*B, and REL), we observed strong correlations (Spearman's rho > 0.6; *p* < 0.05, corrected for multiple testing using the Benjamini–Hochberg FDR) between expression levels and TFRE copy number (Fig. 2). Among these, AP1 demonstrated the highest mCherry expression (rho = 0.916) and did not exhibit saturation even at 12 copies, with the expression level at 12 copies being 1.5-fold higher than at eight copies. The optimal copy number for the other seven TFREs was eight. For instance, a cluster of eight NF*κ*B binding sites resulted in a 2.7-fold increase in expression compared to a cluster with four binding sites, whereas increasing to 12 sites did not further enhance expression compared to eight. The remaining seven TFREs (CaRF, CRE, DRE, EBS1, GABP*β*, GC-box, and SP1) exhibited moderate (0.4 < rho < 0.6, FDR-adjusted *p* < 0.05) or weak (rho < 0.4, FDR-adjusted *p* < 0.05) correlations between expression and copy number. Within this subset, the optimal copy number for DRE was four, whereas it was two for the remaining TFREs.

We categorized all 15 TFREs into three distinct groups based on their expression levels under optimal copies. The strong TFREs, exhibiting expression levels greater than 100 relative to the control, comprised AP1, ERSE, NF*κ*B, and REL. The moderate TFREs, with expression levels ranging from 10 to 100, included AhR/ARNT, ARE, DMP1, DRE, E-box, EBS1, GABP*β*, GC-box, and SP1. Lastly, the weak TFREs, characterized by expression levels below 10, consisted of CaRF and CRE.

### Reporter expression is sensitive to the order and length of TFREs in heterotypic promoters

Heterotypic promoters have been demonstrated to drive stronger reporter expression relative to homotypic promoters^[17]^. Specifically, the expression driven by homotypic promoters, exemplified by the 8× NF*κ*B, was approximately 50% of that achieved by the proprietary CMV promoter. Furthermore, homotypic amplification of TFRE did not further improve promoter strength (Fig. 2). Consequently, to design synthetic promoters with greater strength, we sought to combine diverse TFREs to generate heterotypic promoter constructs.

Smith et al.^[17]^ demonstrated that reporter gene expression was influenced by the spatial arrangement of TFREs in heterotypic promoters. In contrast, Johari et al*.*^[11]^ and Brown et al*.*^[12]^ suggested that TFREs active in homotypic architectures exhibited position-insensitive functionality in heterotypic promoters, thereby supporting a flexible billboard model of promoter regulation. However, all these studies were performed using a transient transfection platform. In this study, we integrated NF*κ*B with six distinct TFREs, spanning a range from strong to moderate, to construct heterotypic promoters arranged in AABB and ABAB configurations. The objective was to assess the impact of TFRE order on heterotypic promoter strength in an SSI platform. Among the six TFRE pairs examined, significant differences in reporter expression were observed in NF*κ*B-AhR/ARNT and NF*κ*B-DMP1 pairs across the two arrangements (Mann–Whitney U test *p* < 0.001). In contrast, the remaining four pairs—NF*κ*B-CaRF, NF*κ*B-GABP*β*, NF*κ*B-SP1, and NF*κ*B-REL—did not show a significant difference (Mann–Whitney U test *p* > 0.1) between the two configurations (Supplementary Fig. S4). These results indicate that alterations in the configuration of heterotypic promoters can modulate reporter expression levels within the SSI context. Consequently, we aimed to design heterotypic promoters with varying strengths by randomly adjusting the order of TFREs while maintaining constant TFRE types and copy numbers.

**Figure 3:**
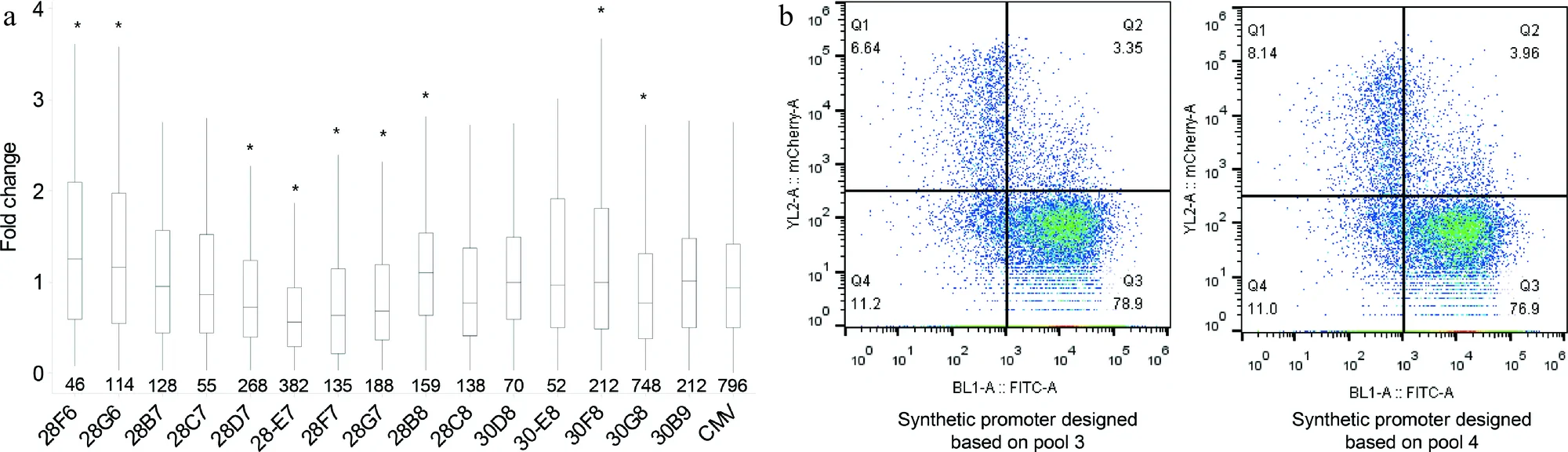
The order and number of TFREs in heterotypic promoters influence reporter expression. (a) Reporter expression levels driven by different synthetic promoters derived from pool 1 and pool 2 were compared to the CMV promoter. Here, '28' or '30' indicates the total number of TFRE copies in each promoter, while 'F6', 'G6', etc., represent serial numbers. Fold changes were calculated by normalizing the MFI of the full CMV promoter. In the box plots, the central rectangle spans the first and third quartiles, the line inside the rectangle represents the median, and the ends of the vertical lines extending beyond the box indicate the minimum and maximum values. Statistical analysis was conducted between the synthetic promoters and the CMV promoter using the Mann–Whitney U test; * *p* < 0.05. The numbers at the bottom of the boxes indicate the number of biological replicates tested. (b) Since the distributions of mCherry expression are nearly identical across all promoters designed based on pool 3 and pool 4, only two exemplar promoters are shown here. The reporter expression of both promoters is log-uniformly distributed.

Four distinct groups of heterotypic promoters were designed based on four pools of TFREs (Supplementary Table S3). A three-bp spacer was included in all heterotypic designs. Pool A included elements from strong (NF*κ*B, REL) and moderate (AhR/ARNT, DMP1, GABP*β*, and SP1) TFRE groups. The CaRF element from the weak TFRE group was added to pool A to create pool B. To further increase the length of the synthetic promoters, we enriched the types of elements in both pool C and pool D. Pool C comprised elements from strong (AP1, ERSE, NF*κ*B, and REL) and moderate (ARE, DRE, E-box, GC-box) TFRE groups. Three elements (EBS1, GABP*β*, and SP1) from the moderate TFRE group, along with CRE from the weak TFRE group, were added to pool C to create pool D. Since TFREs from the strong group were the primary functional elements, we set the copy number to the optimal level found in homotypic promoters (Fig. 2). For elements from the moderate TFRE group, we established the copy number to be less than or equal to their optimal value. For elements from the weak TFRE group, we fixed the copy number at two. In summary, promoters in pool A and pool B had 28 and 30 TFRE copies, respectively, whereas promoters in pool C and pool D each comprised 58 TFRE copies (Supplementary Table S3). We synthesized 10 heterotypic promoters based on pool A and five heterotypic promoters based on pool B by randomizing the order of TFREs. The strength of the 15 heterotypic promoters (sequence information summarized in Supplementary Table S4) was comparable to that of the CMV promoter; however, the strength of each synthetic promoter varied (Fig. 3a). Significant differences (*p* < 0.05) in reporter expression levels driven by different synthetic promoters were frequently observed (Supplementary Table S5), thereby corroborating that the arrangement of TFREs influences promoter strength in SSI. When comparing the reporter expression levels driven by synthetic promoters with those driven by the CMV promoter (Supplementary Table S6), some promoters demonstrated higher activity (e.g., 28F6, 28G6, 28B8, and 30F8), whereas others (e.g., 28E7 and 28F7) exhibited lower activity. Subsequently, 40 heterotypic promoters based on pool C and 18 based on pool D were designed through randomization of TFRE order. Interestingly, the distribution of mCherry reporter expression among these 58 synthetic promoters was nearly identical (Fig. 3b). The expression levels spanned over four orders of magnitude and were log-uniformly distributed in quadrants Q1 + Q4 (Fig. 3b). The underlying mechanism by which promoter length substantially influences the distribution of reporter gene expression remains unknown, but it may be associated with epigenetic silencing^[18,19]^. Accordingly, synthetic promoters containing 28–30 TFREs would be selected for further research.

We selected eight synthetic promoters—28F6, 28E7, 28F7, 28G7, 28B8, 30E8, 30F8, and 30G8—that cover a broad range of reporter expression for stability studies. Both 5' and 3' junction PCRs were conducted to verify the correct integration of the donor sequence in each clonally-derived cell line sorted from mCherry^+^/ZsGreen1^−^ cell populations (Supplementary Fig. S5). The results of digital droplet PCR (ddPCR) indicated that all clonally-derived cell lines harbored a single copy of the mCherry gene (Supplementary Table S7). The MFI of all clonally-derived cell lines did not largely decrease from week 0 (W0) to W9 (Supplementary Table S8). These promoters were deemed capable of continuously and robustly expressing the transgene. Therefore, all eight synthetic promoters were employed in the subsequent study.

### Synthetic promoters can express more msAb product than the CMV promoter in SSI

**Figure 4:**
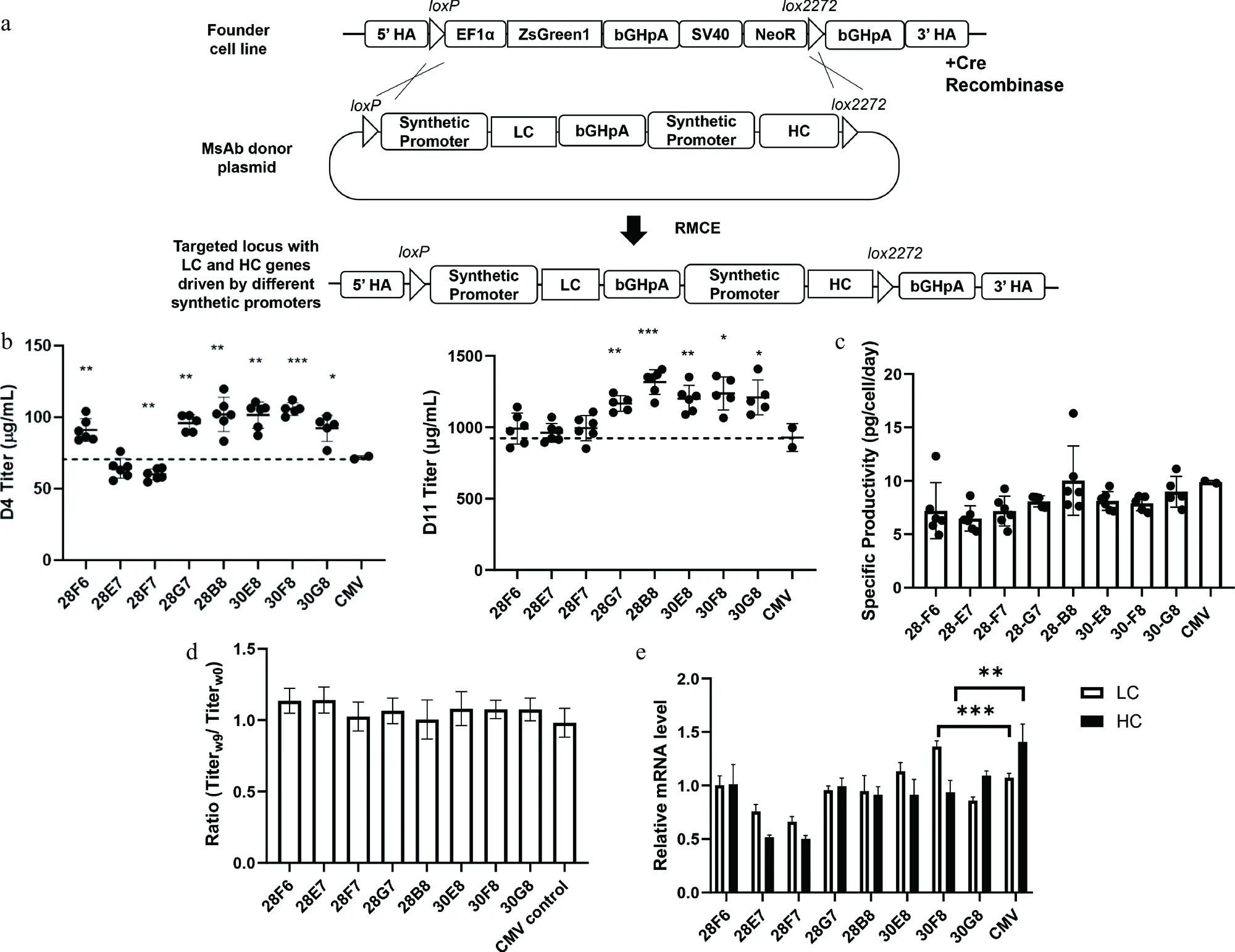
The msAb expression levels driven by eight synthetic promoters were comparable to those of the CMV promoter. (a) Generation of clonally-derived cell lines expressing msAb, correctly integrated and driven by various synthetic promoters. (b) Titers measured on D4 and D11 of fed-batch culture are presented. Dashed lines indicate the average titer value of the CMV control group. Statistical analysis in (b) was performed by the Student's *t*-test comparing the synthetic promoter sample to the CMV promoter sample; * *p* < 0.05, ** *p* < 0.01, *** *p* < 0.001. Sample sizes were *n* = 5 for all synthetic promoters (e.g., 28F6, 28E7, 28F7, 28G7, 28B8, 30E8, 30F8, and 30G8) and *n* = 2 for the CMV promoter. (c) Specific productivity is shown. (d) Stability of msAb titer after 9 weeks of passaging is illustrated. (e) LC and HC gene expression levels during seed train culture were measured by qRT-PCR and normalized to the mean value of 28F6. One tested sample was randomly selected from each group. Error bars represent the standard deviation of three technical replicates (*n* = 3). Statistical analysis in (e) was performed using a Student's *t*-test comparing the synthetic promoter sample to the CMV promoter sample. The result comparing 30F8 and the CMV promoter are shown in the graph. ** *p* < 0.01, *** *p* < 0.001. All *p*-values were calculated and are listed in Supplementary Table S15.

The eight synthetic promoters exhibited comparable or even superior expression capability compared to the CMV promoter when employed to express non-secreting mCherry. To further assess the potential of these eight promoters for secretory msAb expression applications, we positioned each promoter upstream of both light chain (LC) and heavy chain (HC) genes and integrated the entire expression cassette into the target locus via RMCE (Fig. 4a). Following FACS sorting, the expanded clonally-derived cell lines were analyzed using flow cytometry, and their supernatants were utilized for titer assays. Notably, we observed that the proportion of clones exhibiting FITC- and titer > 3 μg/mL (indicating that the cells were secreting antibodies) was 2.7 to 6.5 times greater in the synthetic promoter groups compared to the CMV control group (Supplementary Table S9). This finding suggests that a shorter donor sequence can enhance the RMCE efficiency. The clonally-derived cell lines were further validated by insert PCR (Supplementary Fig. S6) and ddPCR (Supplementary Table S10), and were subsequently subjected to fed-batch culture. At the beginning of the fed-batch process (D4), the titers of 28E7 and 28F7 were approximately 15% lower than those of the CMV promoter. This decrease can be attributed to both LC and HC exhibiting 30% to 50% lower gene transcription levels compared to the CMV promoter (Fig. 4e). In contrast, the titers of other synthetic promoters were higher (Fig. 4b), ranging from 25% to 45% above those of the CMV promoter. By the end of the process (D11), the titers of weaker promoters like 28E7 and 28F7 were conversely ~20% higher than the CMV promoter, while the titers of other synthetic promoters remained 25%–45% higher than the CMV promoter (Fig. 4b). This improvement can be attributed to the enhanced growth of cells using synthetic promoters compared to the CMV promoter. The integrated viable cell densities (IVCD) for cells with synthetic promoters were ~30%–50% higher than those with the CMV promoter (Supplementary Fig. S7), while the specific productivity of the various promoters was comparable to that of the CMV promoter (Fig. 4c). Among all synthetic promoters, the mRNA levels of LC and HC genes during seed train cultures indicated that 28E7 and 28F7 were the weakest, while 30E8 and 30F8 were among the strongest (Fig. 4e). The mRNA level of both LC and HC genes transcribed by 30F8 were 1.8 times higher than 28E7. When calculating the correlation coefficients between mRNA levels and specific productivity (Qp) from D0–D4 (Supplementary Table S11, CMV excluded), we found LC exhibited a higher correlation with Qp (Spearman's rho = 0.833, *p* = 0.010) compared to HC (Spearman's rho = 0.571, *p* = 0.139), indicating productivity was more closely correlated with LC gene expression than with HC gene expression. In addition, the mRNA level of the LC gene from the CMV promoter was around the same level as other synthetic promoters (28F6, 28G7, 28B8, 30G8, 30F8, and 30E8), while the mRNA level of the HC gene was the highest among all promoters. However, the Qp of the CMV promoter was lower than those of the aforementioned synthetic promoters. The discrepancy between mRNA and protein abundance is complex and elusive in this case, suggesting that the mRNA transcripts derived from the CMV promoter may experience different post-transcriptional fates—such as altered nuclear export kinetics, differential localization, or variable resistance to degradation—that ultimately decouple the initial mRNA readout from the final functional protein concentration^[20–22]^. Finally, we repeated the fed-batch culture after passaging the cells for 9 weeks. Since the titer variation range was less than 25% over the 9 weeks, all clonally-derived cell lines were considered stable (Fig. 4d; Supplementary Fig. S8).

### Synthetic promoter combination produced lower levels of bsAb compared to the CMV promoter

**Figure 5:**
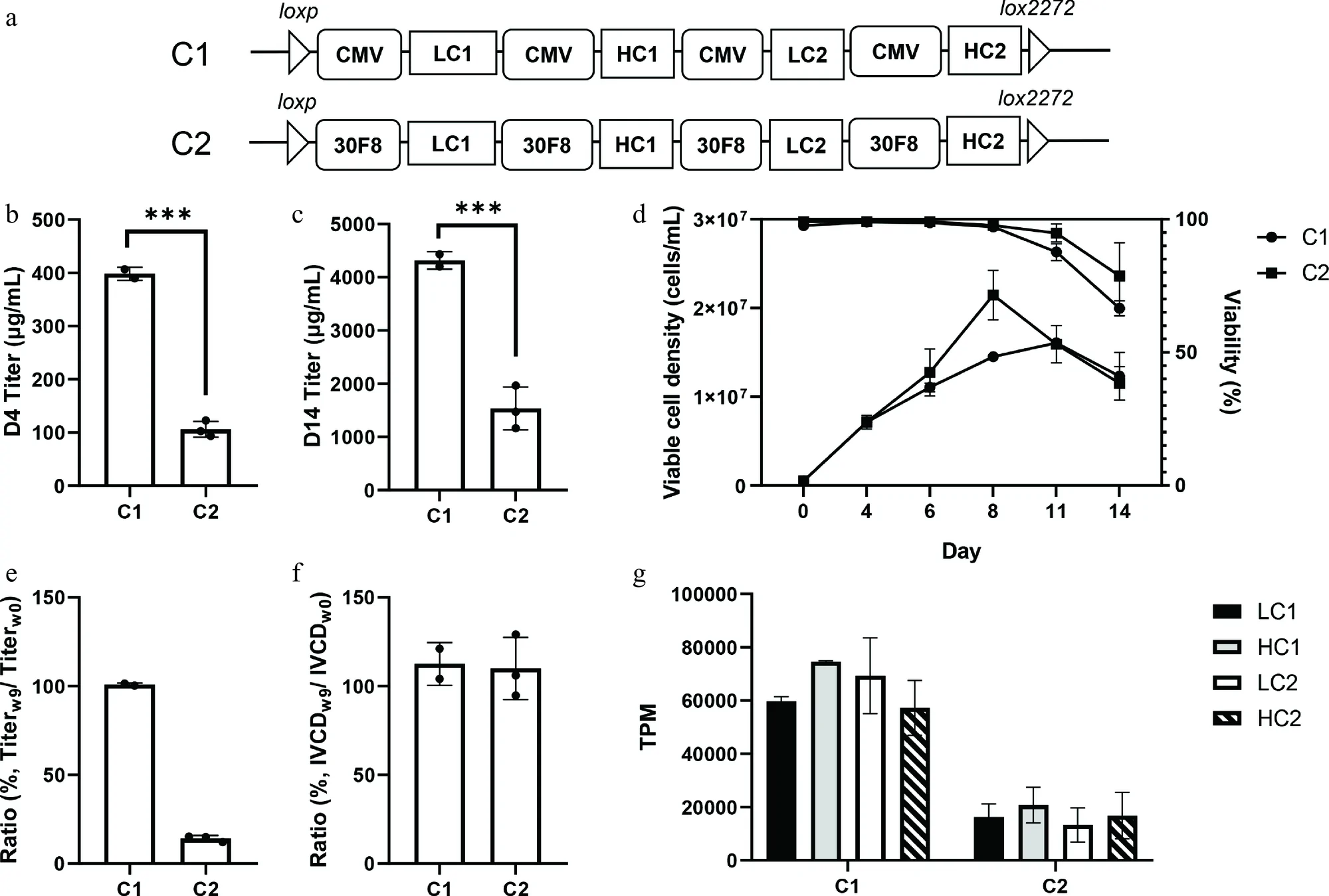
Synthetic promoter combination expresses lower levels of a bsAb than a CMV promoter combination. (a) The CMV or synthetic promoter 30F8 is positioned upstream of each of the bsAb genes. The arrangement of the four bsAb genes is fixed in the order of LC1-HC1-LC2-HC2. A total of two promoter combinations were designed. (b), (c) The fed-batch titers on D4 and D14 are presented. Statistical analysis in (b) and (c) was performed using a Student's *t*-test comparing the C2 to the C1 samples. The results comparing two sets of samples are shown in the graph; *** *p* < 0.001. (d) VCD and viability during the fed-batch process are shown. (e), (f) The stability of fed-batch titer and cell growth over a 9-week passage are presented. (g) Transcription levels of transgenes are measured in transcripts per kilobase million (TPM) for bsAb-producing, clonally-derived cell lines sampled during seed train culture. The error bars represent the standard deviations of three subclones expressing the same transgenes (*n* = 3). In panels (b)–(g), *n* = 2 for C1; *n* = 3 for C2.

We further applied the newly discovered promoter in the expression of bsAb using the most potent synthetic promoter, 30F8. Four genes encoding bsAb^[23]^ were concatenated in a fixed topology to construct a four-in-one donor plasmid. Either the CMV promoter or the 30F8 promoter was positioned upstream of each of the four genes, resulting in the generation of constructs C1 and C2 (Fig. 5a; C1 and C2), respectively. During the screening of clonally-derived cell lines, we found that the proportion of clones with FITC- and titer > 3 μg/mL in C2 was 10-fold greater than that in C1 (Supplementary Table S12). This finding further supports the notion that shorter sequence length facilitates RMCE (Supplementary Table S9). An alternative explanation for this observation is that expression of bsAb genes driven by the CMV promoter may be generally detrimental to cellular viability, thereby reducing recovery rates. All clonally-derived cell lines were verified through multiple assays including insert PCR (Supplementary Fig. S9) and ddPCR (Supplementary Table S13) before being subjected to fed-batch culture. Finally, we obtained two clonally-derived cell lines for C1 and three clonally-derived cell lines for C2.

After a 14-d fed-batch culture, the CMV promoter combination expressed more products than the pure synthetic promoter combination in an integrated four-gene cassette (Fig. 5b, c). This observation contrasts with the results from the msAb (two-gene cassette), where synthetic promoters produced greater final product yields than the CMV promoter (Fig. 4b). Notably, the IVCD of the clonally-derived cell line in C1 was ~20% lower than C2 (Fig. 5d), which was consistent with the msAb finding (Fig 4b). Following 9 weeks of passaging, we found the titer value of C2 was ~20% of that at week 0 (W0), whereas C1 maintained stable titers (Fig. 5e). IVCD remained unchanged in both combinations (Fig. 5f). We further used RNA-seq to quantify the transcription levels of the four genes in the two promoter combinations (Fig. 5g). The transcription level of all four genes in C2 was only ~30% of that in C1, which explains the decrease in titer values.

## Discussion

Recent studies in human and *Drosophila* cells have demonstrated that the results from plasmid-based transcriptional regulatory elements are highly correlated with those from genome-integrated ones, indicating the absence of genomic context may have a less significant impact on promoter strength than previously assumed^[24,25]^. In the present study, the correlation coefficient for the expression of mCherry driven by the top TFREs—including REL, NF*κ*B, AP1, NF*κ*B-p65, ERSE, DRE, RELA, GABP*β*, USF1, and ARE—between SSI and transient platforms was calculated to be 0.598 (Pearson's *r*). This result indicates that the locus substantially influences promoter strength. Interestingly, when comparing our non-secreting reporter data (mCherry) from both transient and SSI platforms with previously published secreted alkaline phosphatase (SEAP) data^[11]^ obtained from the transient platform (Supplementary Table S14), a higher correlation coefficient was observed for the SEAP-transient/mCherry-transient pair (Pearson's *r* = 0.90) than for the SEAP-transient/mCherry-SSI pair (Pearson's *r* = 0.84). These findings imply that the SSI platform exhibits distinct characteristics relative to the transient platform in the evaluation of promoter strength. Consequently, a landing pad locus-specific promoter may be more appropriate for the SSI platform, which can facilitate the design of more customized synthetic promoters. To date, large-scale promoter studies have predominantly utilized transient expression platforms, assessing either mRNA levels^[26]^ or reporter levels^[27]^. The SSI approach presents a novel methodology for studying genomic regulatory elements and advances our comprehension of the relationship between genomic context and promoter strength.

Although our heterotypic promoters consist of ~30 TFREs spanning a total length of 500 bp—one-third the length of the ~1,500 bp CMV promoter (noting some versions of the CMV promoter are shorter than the one used in this study^[11]^)—the strength of these synthetic promoters is generally comparable to that of the proprietary CMV promoter. Endogenous promoters typically harbor diverse cis-regulatory modules, including silencers, insulators, and tethering elements, in addition to promoter-proximal elements and enhancers. These redundant elements may exert inhibitory effects on gene expression^[28]^. In contrast, synthetic promoters are devoid of these redundant regulatory components, thereby enabling more efficient modulation of recombinant transcriptional activity^[11]^. Additionally, the use of longer endogenous promoters can lead to the titration of transcription factors away from endogenous genes, consequently altering their expression profiles. As a result, the functional potency of longer promoters, such as the CMV promoter, may be effectively constrained by their broader impact on cellular physiology^[29,30]^. This is evident in our study by the observed significant reduction in cell growth following integration of transgene cassettes into the FCL genome (Supplementary Fig. S7, Fig. 5d). We can further enhance the strength of current synthetic promoters by optimizing their length or by adjusting the arrangement of TFREs. With regard to the former, we can reduce the copy number of TFREs for promoters from pool C and D (currently containing 58 TFREs) to achieve a more focused reporter expression level, or to add new types of TFREs for promoters from pool A and B to further improve reporter expression level. With regard to the latter, application of library screening is necessary to select even stronger promoters, given that the arrangement of TFREs critically influences promoter strength. The extensive datasets generated through such screening can also be used to train machine learning models to design even stronger promoters *in silico*^[26,27]^. These newly designed strong promoters hold potential applications in both SSI and random integration (RI) methodologies to further elevate cellular expression levels.

The BsAb titer driven by the CMV promoter combination C1 was observed to be higher than that driven by C2, a finding that contrasts with the results obtained for mCherry (Fig. 3a) and msAb (Fig. 4b). Additionally, a significant decline in BsAb production driven by C2 was detected after 9 weeks of passaging (Fig. 5e). In contrast, the synthetic promoter combination (30F8) demonstrated stable expression of msAb (Fig. 4e). Moreover, the mRNA levels of both LC and HC genes driven by 30F8 were comparable to those driven by CMV when expressing msAb (Fig. 4e). However, there was a markedly reduction in the transcription levels of the four genes for C2 (Fig. 5g) compared to C1, indicating that the current synthetic promoter combinations may be more susceptible to promoter-promoter interference^[11,31]^ than CMV promoter combinations in a four-gene cassette, as opposed to a two-gene cassette. To be more specific, as some TFREs respond to intracellular cues^[32] ^(e.g., nutrient limitation or ER stress) that fluctuate during the changing environment of a fed-batch process, more clustered promoters may be more susceptible to this throughout the bioprocess. Since SSI can largely minimize the variation between clones^[6]^, the limited number of clones can effectively reflect the expression pattern of bsAb using these two different promoter combinations. Undoubtedly, having more clones for both C1 and C2 would increase confidence in the observed differences between C1 and C2. The CMV promoter encompasses at least 12 distinct TFRE types^[33]^ as well as multiple redundant elements such as introns, making it considerably more diverse than the current set of synthetic promoters. When four promoters are closely positioned, CMV promoters are more likely to coordinate the activity of different components to function harmoniously compared to shorter synthetic promoters. Furthermore, the 3-bp spacers within synthetic promoters may be insufficient, and such complexities in promoter architecture are generally underexplored in synthetic constructs reported in the literature. It is also important to note that a bsAb requires a more sophisticated folding process and is therefore fundamentally different from a msAb, which likely contributes to differences in productivity. To increase titer levels, we can design new synthetic promoters using distinct TFRE types such as AP1, ERSE, and GC-box or use natural promoters in combination with existing synthetic promoters to enhance the complexity of promoter combinations. Alternatively, we can divide the current four-gene cassette into two independent two-gene cassettes and integrate them at two separate loci to reduce interference. Additionally, promoter-promoter interference was also observed in the msAb testing, although to a lesser extent than observed with bsAb. For example, the 30F8 promoter demonstrated a higher expression of the LC gene but a lower expression of the HC gene relative to the CMV promoter (Fig. 4e). By employing promoters from different sources, we can minimize such interference, thereby increasing the final titer of msAb. Furthermore, we expressed another msAb driven by either the 30F8 or 28E7 promoter, respectively, during the mini-pool phase using a random integration method. The results indicated that the expression level driven by the 30F8 was ~2.0 g/L while that driven by the 28E7 promoter was ~1.2 g/L. The former expression level is significantly higher than the latter (specific data not shown). These results suggest that the synthetic promoters characterized in this study have the potential for application in random integration or other loci.

As SSI technology has gained prominence in the domain of cell line development, addressing its inherent limitations has emerged as a critical research topic for biopharmaceutical companies. Utilizing stronger landing pad locus-specific synthetic promoters presents a more straightforward strategy compared to increasing the copy number of the inserted gene, which involves much more complex DNA cloning procedures. Furthermore, optimizing the expression levels of individual genes within complex molecular constructs is more achievable through various combinations of synthetic promoters than through gene rearrangement strategies^[34]^, which require testing of multiple transgene configurations^[35]^. Therefore, design and application of landing pad locus-specific synthetic promoters are poised to become essential tools for the ongoing enhancement of the SSI platform.

## Materials and Methods

### Plasmid design and construction

Landing pad plasmids designed for SSI were previously incorporated into the CHO genome at the Nfat5 locus on Chromosome 3 to establish the founder cell line (FCL). The landing pads comprised several critical elements, including a 5′ homology arm for targeted insertion, *loxP* and *lox2272* recombination sites, EF1*α* promoter, ZsGreen1 gene, bGH poly(A) tail, SV40 promoter, NeoR gene, bGH poly(A) tail, and a 3′ homology arm. The fluorescence donor plasmids harbored an mCherry gene along with other genetic elements, including an enhancer composed of multiple TFREs, a CMV core promoter, a bGH poly(A) tail, an SV40 promoter, and a puromycin resistance (puroR) gene. These elements were flanked by a *loxP* site at the 5' end and a *lox2272* site at the 3' end. Enhancer sequences were synthesized and cloned into SpeI and NotI restriction sites between the *loxP* and the CMV core promoter (GenScript) to generate functional promoters. Features of both landing pads and mCherry RMCE donor plasmids are illustrated in Fig. 1a and detailed in Supplementary Tables S2 and S4. Fragments bearing msAb LC and HC genes or bsAb LC1, HC1, LC2 and HC2 were synthesized and cloned into a vector containing *loxP* and *lox2272* sequences (GenScript). Various enhancer-CMV core promoters or full CMV promoter sequences were synthesized (GenScript) and cloned upstream of the LC or the HC genes, respectively. All constructs were validated by Sanger sequencing (GenScript) and were purified using NucleoBond Xtra Midi EF (Macherey-Nagel). Detailed sequence information of all LC and HC genes is provided in Supplementary Table S4. The Cre-recombinase-expressing plasmid (Sigma-Aldrich, OG591) was used for all the RMCE processes.

### mCherry expression cell line generation by RMCE and fluorescent level analysis

Cells of FCLs derived from CHO-K1, at a density of 1E6/mL, were transfected with an mCherry RMCE donor plasmid and a Cre-recombinase plasmid in a 3:1 (w:w) ratio using polyethylenimine (Yeasen). Transfected cells were cultured in 96-half-deepwell microplates (Enzyscreen) containing CD CHO (Gibco) medium supplemented with 8 mM L-glutamine (Gibco). Plates were maintained in a humidified incubator set to 37 °C with 5% CO_2_ and agitated at 800 rpm. Cells were passaged for a week and were selected with 3 μg/mL puromycin (Gibco) for an additional 10 d. After pool selection, cells were analyzed using an Attune NxT flow cytometer (Thermo Fisher Scientific), assessing mCherry fluorescence intensity in ~50,000 individual cells.

### Antibody expression cell line development by RMCE

A total of 1 × 10^7^ FCLs were transfected with 30 μg antibody donor plasmids and 10 μg Cre-recombinase plasmids using the Gene Pulser Xcell (Bio-Rad). After transfection, the cells were passaged in a 125 mL shake flask vessel at 120 rpm, 36.5 °C, and 6% CO_2_ for 1 week. Subsequently, single-cell sorting was performed to isolate the recombined cell population.

### Bulk and single-cell sorting

To isolate correctly integrated cells following RMCE, both bulk and single-cell sorting were performed on a FACSAria Fusion (BD) equipped with 488 nm and 561 nm lasers. Untransfected FCLs were used as controls to establish gating parameters for identifying ZsGreen1-negative or mCherry-positive populations. Sorted cells were seeded in flat-bottom 96-well plates (Corning) containing 200 μL of in-house cell cloning medium supplemented with 8 mM L-glutamine. Plates were cultured at 36.5 °C in a humidified 6% CO_2_ in air (v/v) incubator. After 14 d of culture, cells initially seeded in 96-well plates were expanded sequentially into 24-well plates (Corning), followed by 24-deep-well plates (Satorius), and subsequently transferred to 125 mL Erlenmeyer flasks (Corning) for further growth.

### Confirmation of correct integration by PCR

Genomic DNA (gDNA) was extracted from the cell pellet using the TIANamp genomic DNA kit (Tiangen) or MagPure Universal DNA LQ kit (Magen) according to the manufacturer's instructions. For 5′ and 3′ junction PCRs, 1 μL of gDNA was used as template for PCR using Platinum II hot-start green PCR master mix (2×) (Thermo Fisher Scientific) following the manufacturer's instructions. For insert PCR, 1 μL of gDNA was used as template for PCR using PrimeSTAR GXL DNA polymerase (Takara) following the manufacturer's instructions. PCR products were visualized on 1% agarose gels. PCR primers for 5′/3′ junction and insert PCRs are listed in the Supplementary Table S4.

### Copy number analysis by ddPCR

Copy numbers of mCherry, LC, and HC genes were quantified by droplet digital PCR (ddPCR) using the QX200 system (Bio-Rad). The optimal dilution fold of template was established based on a preliminary qPCR result. Each ddPCR reaction, with a total volume of 20 μL, comprised 10 μL of ddPCR Supermix (Bio-Rad), two primer sets (1.8 μL, each at 10 μM), two probes (0.5 μL, each at 10 μM), and 1 μL of diluted template. One primer/probe set was specific to the target gene (mCherry gene, HC gene, or LC gene), while the other set was specific to an endogenous CHO gene, C1GALT1C1 (COSMC). The COSMC gene was used as the internal control gene for normalization purposes in the duplex assay, as it was previously determined to have one copy per cell^[1]^. The PCR thermal cycling conditions were 10 min at 95 °C, followed by 40 cycles of 94 °C for 30 s and 60 °C for 1 min, then 98 °C for 10 min to deactivate the enzyme. After PCR, the droplets were read on a QX200™ Droplet Reader (Bio-Rad). The data were collected and analyzed using QuantaSoft software (Bio-Rad).

### Long-term cell culture

Clonally-derived cell lines were maintained in CD CHO medium supplemented with 8 mM L-glutamine and cultivated in 125 mL Erlenmeyer flasks. Cells were incubated at 37 °C, 5% CO_2_, at 120 rpm and were passaged every 3–4 d at a seeding density of 5 E5 cells/mL. The entire cell passaging lasted for 9 weeks.

### Fed-batch culture

Fed-batch production cultures were conducted in shake flasks with proprietary chemically defined production media supplemented with 8 mM L-glutamine. Cells were seeded at 5 × 10^5^ cells/mL in a 20 mL volume on day 0. Cultures received 1, 1, and 1.5 mL of Efficient-Pro™Feed 1 medium (Gibco), and 0.4, 0.4, and 0.6 mL of 200 mM L-glutamine on days 4, 6, and 8, respectively. Viable cell density and viability of the cells in culture were assessed on days 0, 4, 6, 8, and 11 using a Countstar Bio Tech instrument (CountStar). Glucose concentrations were measured on days 4, 6, and 8 using a Biosen C-Line Glucose and Lactate analyzer (EKF Diagnostic). An appropriate amount of glucose was added to maintain the final glucose concentration at 12 g/L. Titers were quantified using an Octet QKe system (Fortebio) with a Protein A sensor (Fortebio).

### Relative mRNA level measured by real-time qPCR

Total RNA was extracted from cells using the RNA Easy Fast Cell kit (Tiangen). cDNA was synthesized from ~1 μg of total RNA using the FastKing RT Kit (with gDNAse, Tiangen) for qPCR. qPCR assays were conducted on a LightCycler 480 Instrument II (Roche) using TaqMan Fast qPCR Master Mix (Roche) in a duplex format, targeting GOI and one internal control gene. The thermal cycling conditions were 94 °C for 3 min; 45 ×: 94 °C for 5 s, 57 °C for 15 s, 72 °C for 30 s. Sequences of probes and primers for both the GOIs and the internal control gene are listed in Supplementary Table S4. Relative mRNA expression levels of GOIs were quantified using the ΔΔCt method^[36]^.

### RNA extraction for RNA-seq

A total of 1 × 10^7^ cells were harvested during the seed train culture. The cells were centrifuged at 1,000 g for 5 min, after which the supernatant was carefully removed. Total RNA was isolated from the cell pellets using TRIzol® Reagent in accordance with the manufacturer's instructions (Invitrogen). Genomic DNA was removed using DNase I (TaKara). The integrity and quality of RNA were assessed using a 2100 Bioanalyser (Agilent), while quantification was performed with an ND-2000 instrument (NanoDrop Technologies). High-quality RNA (OD260/280 = 1.8~2.2, OD260/230 ≥ 2.0, RIN ≥ 6.5, 28S:18S ≥ 1.0, > 10 μg) was used to construct a sequencing library.

### Library preparation and Illumina HiSeq sequencing

RNA-seq transcriptome libraries were constructed using a TruSeq^TM^ RNA sample preparation Kit (Illumina). Messenger RNA was isolated through oligo(dT) bead selection and fragmented using fragmentation buffer. cDNA synthesis, end repair, A-base addition, and ligation of the Illumina-indexed adaptors were performed according to the manufacturer's instructions. The libraries were then size-selected to enrich for cDNA fragments ranging from 200 to 300 bp on a 2% agarose gel, followed by PCR amplification for 15 cycles using Phusion DNA polymerase (NEB). Paired-end libraries were sequenced on an Illumina NovaSeq 6000 with about 20 million reads per sample (150 bp * 2, Shanghai BIOZERON Co., Ltd).

### Read-quality control and mapping

The raw paired-end reads underwent trimming and quality control using Trimmomatic with parameters SLIDINGWINDOW:4:15 MINLEN:75 (v0.36 www.usadellab.org/cms/uploads/supplementary/Trimmomatic). Subsequently, the clean reads were separately aligned to the *Cricetulus griseus* (Chinese hamster) representative genome assembly CriGriPICRH1.0 (GCF_003668045.3) serving as the reference, using hisat2 (https://ccb.jhu.edu/software/hisat2/index.shtml) software with default parameters. The transgenes (LC1, HC1, LC2, and HC2) coding sequences used in this study were all incorporated into the transcriptome reference. Quality assessment of these data was performed using qualimap_v2.2.1 (https://qualimap.bioinfo.cipf.es/). We used htseq (https://htseq.readthedocs.io/en/release_0.11.1/) to count each gene read.

## SUPPLEMENTARY DATA

Supplementary data to this article can be found online.

## Data Availability

The data that support the findings of this study are available from the corresponding author upon reasonable request.
