# Supplementary material for: Synthetic promoters developed through a site-specific integration system enhance antibody expression in fed-batch culture
Source: Eng Life Sci. 2026 Jul 24;26(7):e005. doi: 10.48130/els-0026-0004 (PMC13599642; doi:10.48130/els-0026-0004)

Fig. S1: Proof-of-principle RMCE experiment with different mCherry constructs confirmed functionality of puromycin enrichment and poly(A) trap. A: Tunable expression patterns were generated depending on promoter type preceding mCherry. The MFI of mCherry in Q1 of each panel indicates strength of the promoter. B: Target band size by 5' junction PCR was 1-2 kb or over 2kb (CMV) depending on promoter size; target band size by 3' junction PCR was ~1 kb.

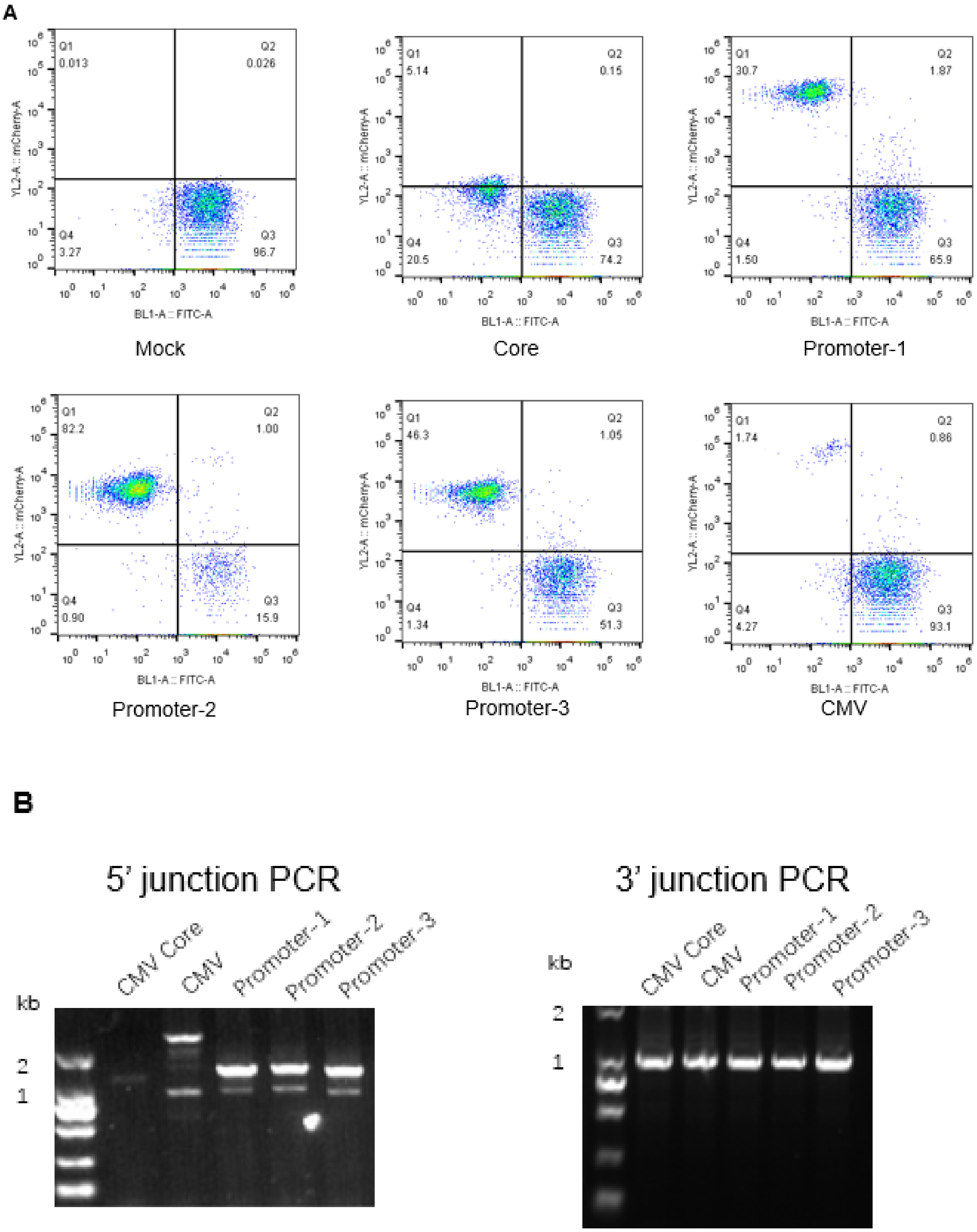

Supplement: Supplementary file 1 [file els-2026-26-7-0004-S1.zip › 10.48130_els-0026-0004-Suppl-FigureS1.pdf]
