# Supplementary material for: Synthetic promoters developed through a site-specific integration system enhance antibody expression in fed-batch culture
Source: Eng Life Sci. 2026 Jul 24;26(7):e005. doi: 10.48130/els-0026-0004 (PMC13599642; doi:10.48130/els-0026-0004)
Supplement: Supplementary file 1 [file els-2026-26-7-0004-S1.zip › 10.48130_els-0026-0004-Suppl-FigureS2.pdf]

Fig. S2: Schematic diagram of the junction and out-out PCR amplification ranges.

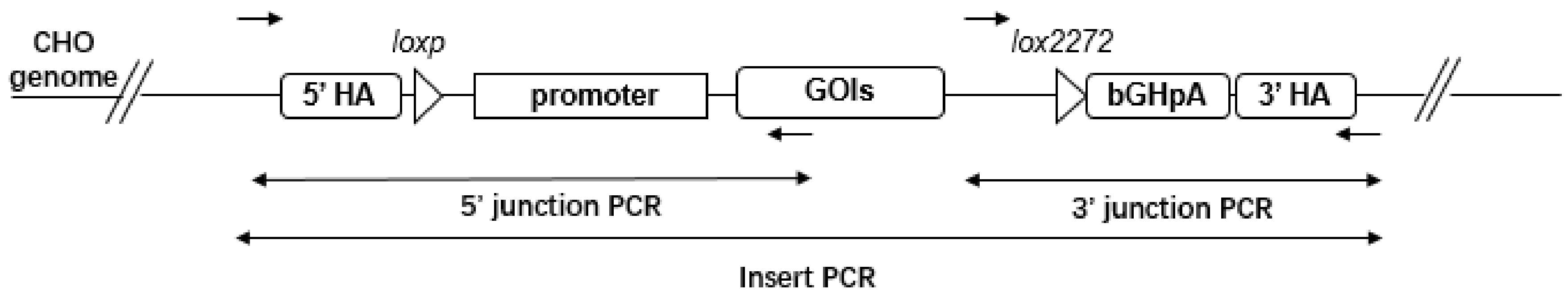

Fig. S2: Proof-of-principle RMCE experiment with different mCherry constructs confirmed functionality of puromycin enrichment and
