# Supplementary material for: Synthetic promoters developed through a site-specific integration system enhance antibody expression in fed-batch culture
Source: Eng Life Sci. 2026 Jul 24;26(7):e005. doi: 10.48130/els-0026-0004 (PMC13599642; doi:10.48130/els-0026-0004)

Fig. S3: Screening of homotypic promoters (A) Eight copies of each TFRE were positioned upstream of a CMV core promoter in a reporter vector encoding mCherry. The fold change was calculated by normalizing with MFI of the full CMV promoter. (B) MFIs of mCherry driven by different homotypic promoters in transient condition. Fold change was calculated by normalizing with MFI of full CMV promoter.

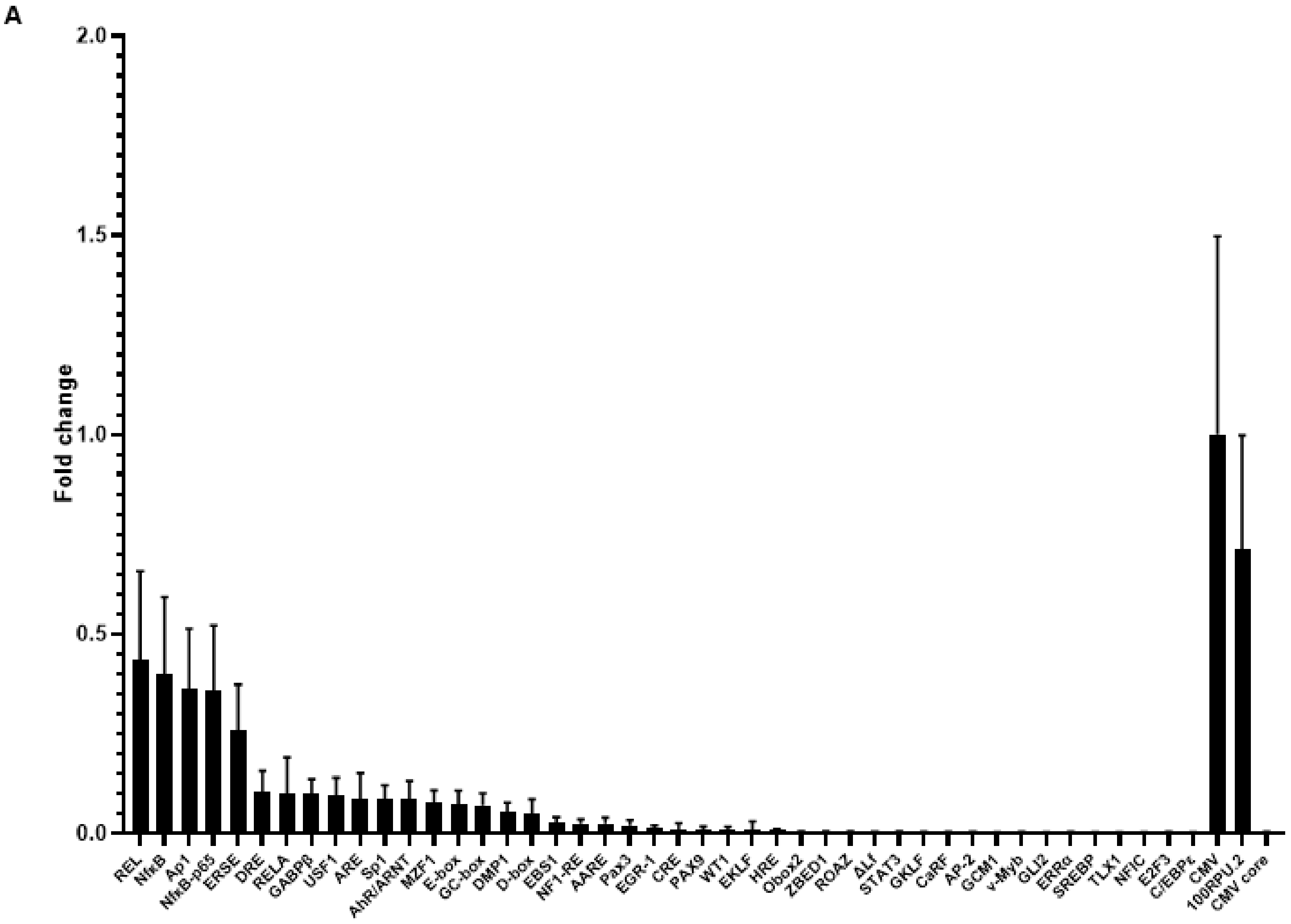

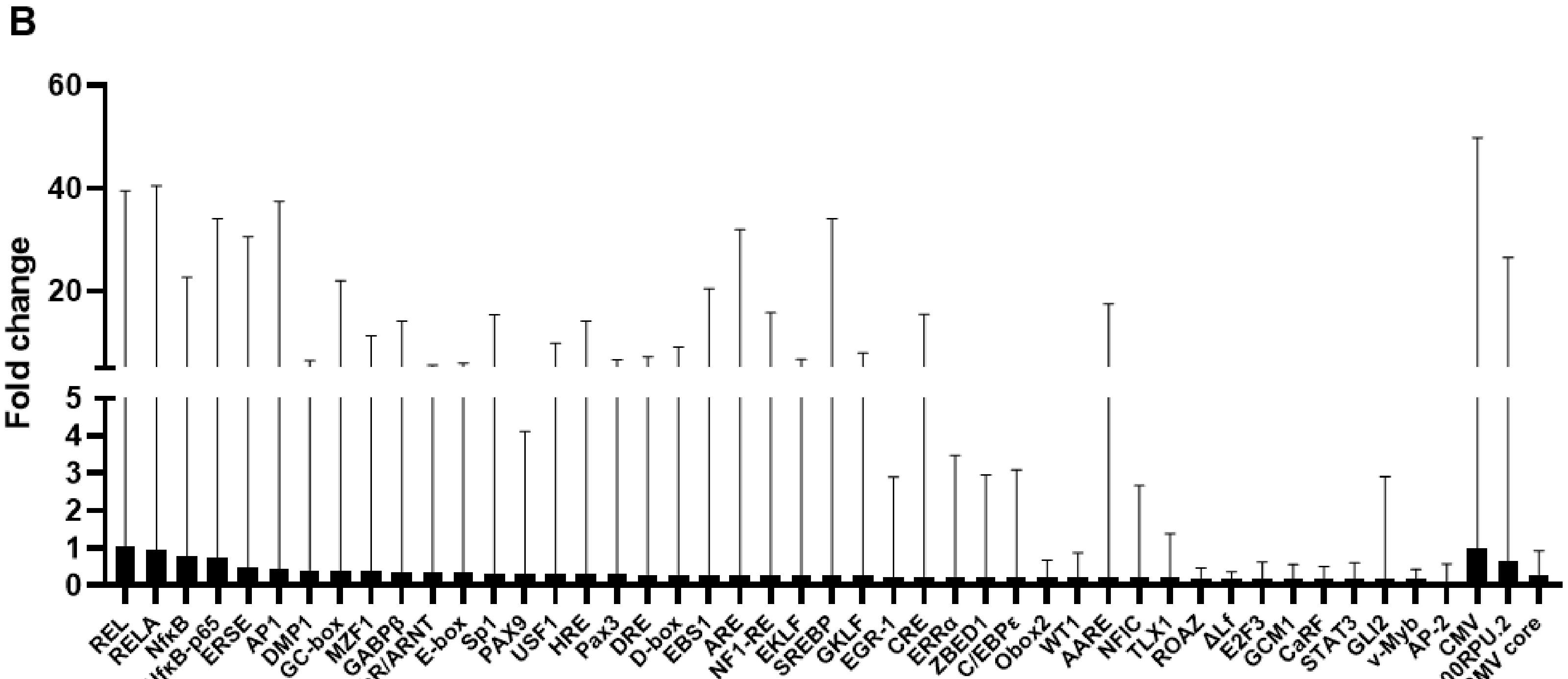

Supplement: Supplementary file 1 [file els-2026-26-7-0004-S1.zip › 10.48130_els-0026-0004-Suppl-FigureS3.pdf]
