# Supplementary material for: Synthetic promoters developed through a site-specific integration system enhance antibody expression in fed-batch culture
Source: Eng Life Sci. 2026 Jul 24;26(7):e005. doi: 10.48130/els-0026-0004 (PMC13599642; doi:10.48130/els-0026-0004)

Fig. S4: A: Direct comparisons of reporter expression between AABB and ABAB arrangements. A was fixed to dual NFκB, while B could be dual AhR/ARNT, CaRF, DMP1, GABPβ, SP1 or REL. Two combinations (NFκB-AhR/ARNT and NFκB-DMP1) were identified to express reporter gene differently in two arrangements (Mann-Whitney U test, ***$p \leq 0.001$). In the box plots, the central rectangle spanned the first and third quartiles, the line inside the rectangle was the median, and the ends of vertical lines beyond the box indicated the minimum and maximum values. The numbers at the top of the boxes represent the number of tested samples.

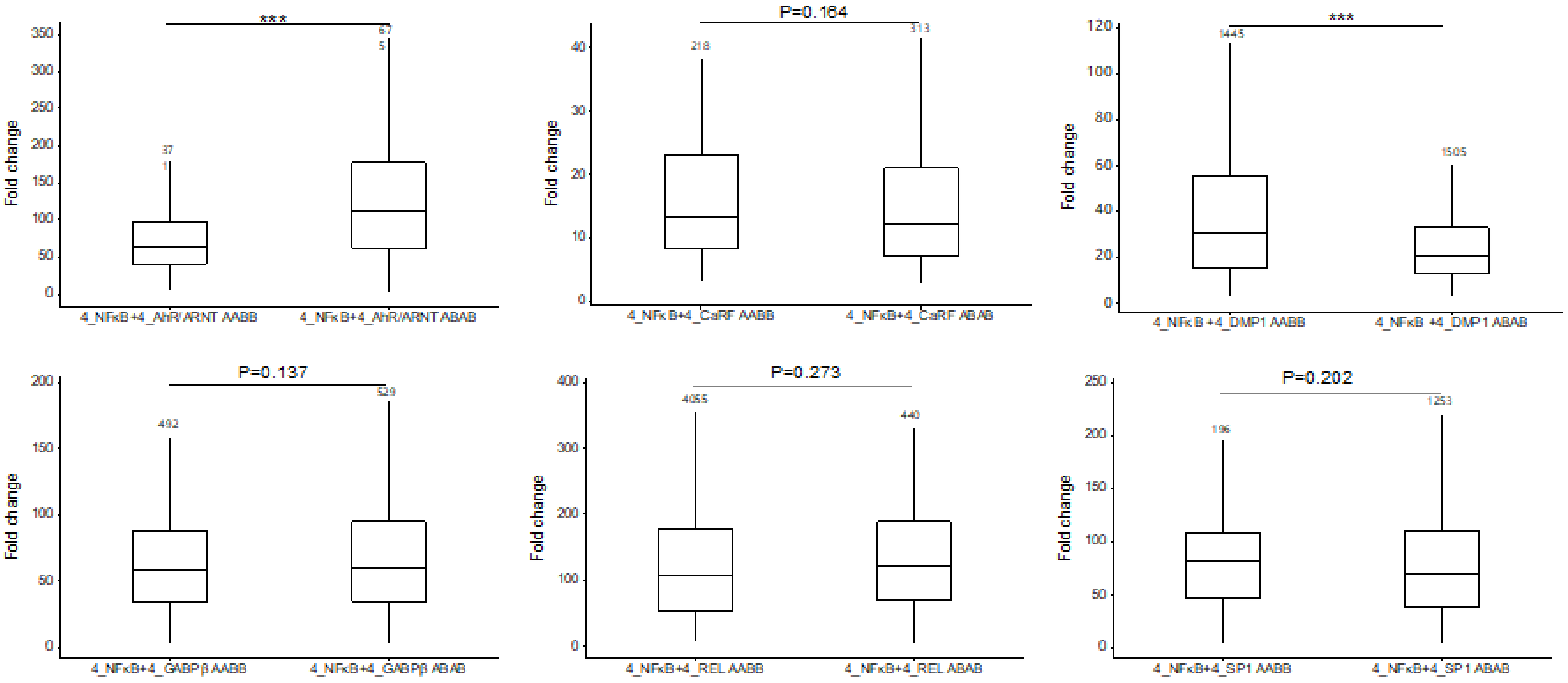

Supplement: Supplementary file 1 [file els-2026-26-7-0004-S1.zip › 10.48130_els-0026-0004-Suppl-FigureS4.pdf]
