# Supplementary material for: Synthetic promoters developed through a site-specific integration system enhance antibody expression in fed-batch culture
Source: Eng Life Sci. 2026 Jul 24;26(7):e005. doi: 10.48130/els-0026-0004 (PMC13599642; doi:10.48130/els-0026-0004)

Fig. S7: (A) Viable cell density (VCD) and viability during the fed-batch process at W0 are shown. The error bars represent the standard deviation of all subclones. (B) Calculation of integrated viable cell density (IVCD) driven by various promoters.

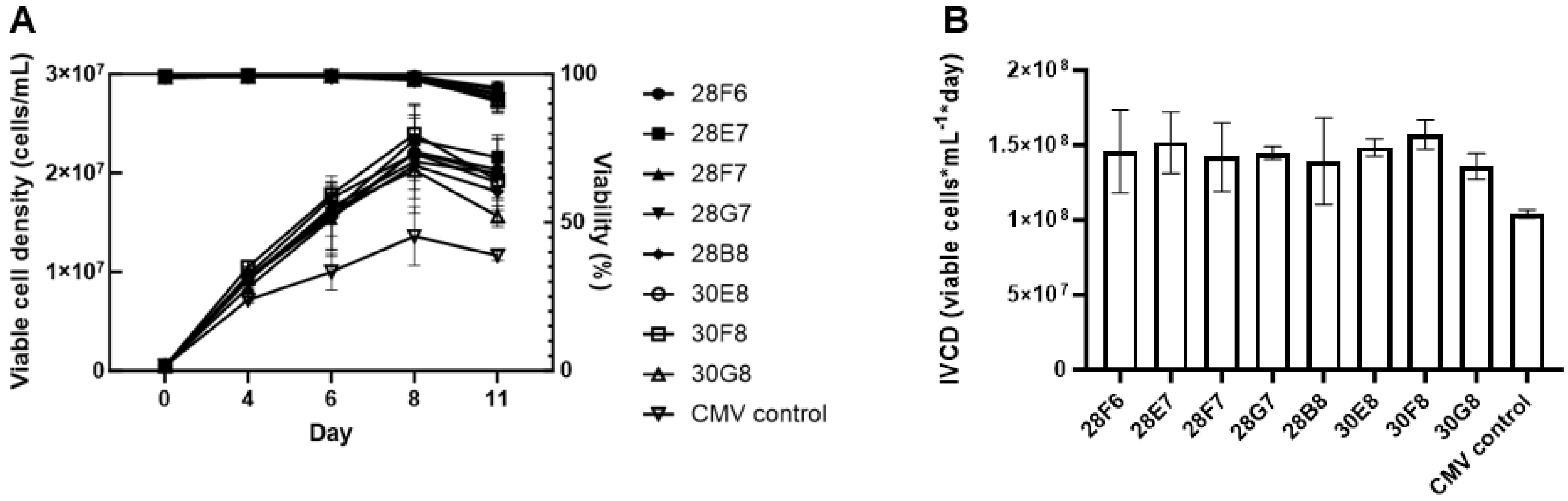

Supplement: Supplementary file 1 [file els-2026-26-7-0004-S1.zip › 10.48130_els-0026-0004-Suppl-FigureS7.pdf]
