# Supplementary material for: Synthetic promoters developed through a site-specific integration system enhance antibody expression in fed-batch culture
Source: Eng Life Sci. 2026 Jul 24;26(7):e005. doi: 10.48130/els-0026-0004 (PMC13599642; doi:10.48130/els-0026-0004)

Fig. S8: Eight synthetic promoters maintained stable msAb expression in W9. A: After 9 weeks of passage, all monoclonal cells were cultivated in 11-day fed-batch mode. Dashed lines represent the average titer value of CMV control group. B: VCD and viability during fed-batch process. The error bars represent standard deviation of all biological replicates.

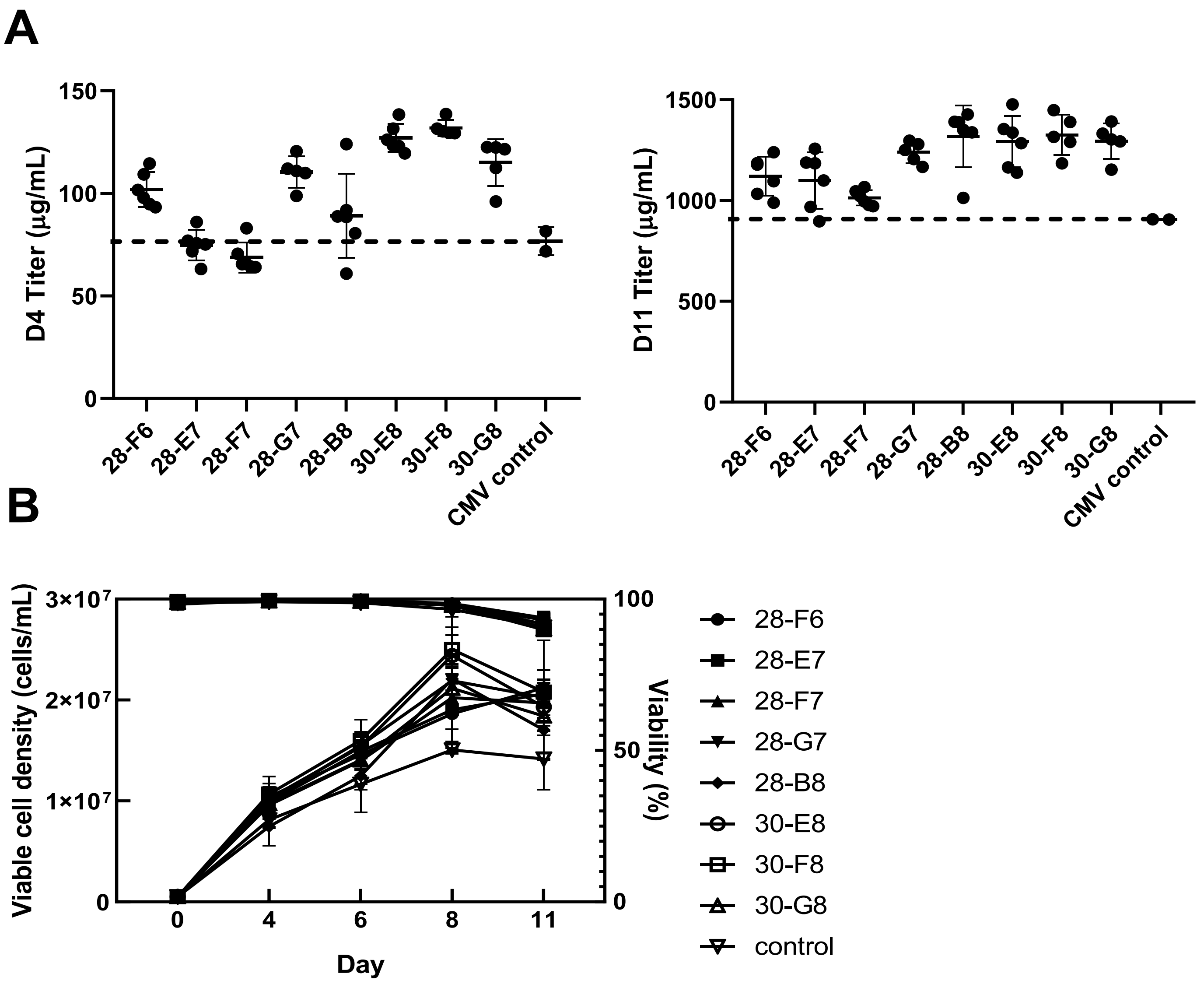

Supplement: Supplementary file 1 [file els-2026-26-7-0004-S1.zip › 10.48130_els-0026-0004-Suppl-FigureS8.pdf]
