# Supplementary material for: Synthetic promoters developed through a site-specific integration system enhance antibody expression in fed-batch culture
Source: Eng Life Sci. 2026 Jul 24;26(7):e005. doi: 10.48130/els-0026-0004 (PMC13599642; doi:10.48130/els-0026-0004)

Fig. S9: Verification of correct integration of bsAb genes with different promoter combinations. Target band size by out-out PCR was ~12 kb for C1 and 9 kb for both C2.

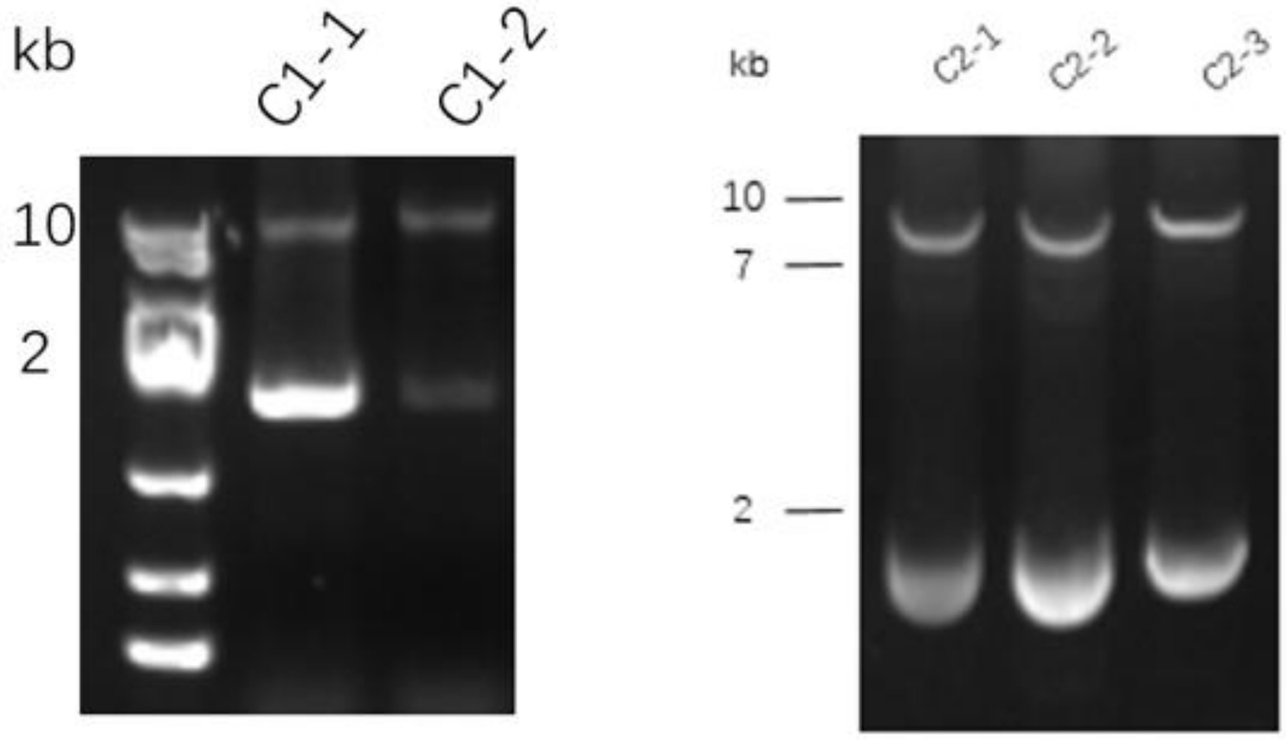

Supplement: Supplementary file 1 [file els-2026-26-7-0004-S1.zip › 10.48130_els-0026-0004-Suppl-FigureS9.pdf]
