# Supplementary material for: Synthetic promoters developed through a site-specific integration system enhance antibody expression in fed-batch culture
Source: Eng Life Sci. 2026 Jul 24;26(7):e005. doi: 10.48130/els-0026-0004 (PMC13599642; doi:10.48130/els-0026-0004)
Supplement: Supplementary file 1 [file els-2026-26-7-0004-S1.zip › 10.48130_els-0026-0004-Suppl-TableS1.pdf]

Supplementary Material

Table S1: Copy number of mCherry expressed by different promoters

Clone ID	mcherry (FAM) 1 μL DNA final copies	COSMC (VIC) 1 μL DNA final copies	Target-reference ratio (mCherry copy #)
Core	5840	5280	1.11
CMV	4680	4160	1.13
Promoter-1	5240	5200	1.01
Promoter-2	4240	3760	1.13
Promoter-3	2740	2420	1.13
