# Supplementary material for: Synthetic promoters developed through a site-specific integration system enhance antibody expression in fed-batch culture
Source: Eng Life Sci. 2026 Jul 24;26(7):e005. doi: 10.48130/els-0026-0004 (PMC13599642; doi:10.48130/els-0026-0004)
Supplement: Supplementary file 1 [file els-2026-26-7-0004-S1.zip › 10.48130_els-0026-0004-Suppl-TableS10.pdf]

Table S10: Copy number of LC and HC genes of msAb in different monoclonal cells. Ratio between 0.5 to 1.5 is considered as single copy. Samples highlighted with gray was not subject to subsequent experiments.

Clone ID	LC (FAM) 1 μL DNA final copies	COSMC (VIC) 1 μL DNA final copies	Target-reference ratio (LC copy #)
28F6-1	11280	10520	1.07
28F6-2	18840	16440	1.15
28F6-3	8700	7600	1.14
28F6-4	14520	14960	0.97
28F6-5	29400	25320	1.16
28F6-6	15520	14040	1.11
28E7-1	10160	10360	0.98
28E7-2	11440	11000	1.04
28E7-3	8600	7600	1.13
28E7-4	15420	12660	1.22
28E7-5	17820	15660	1.14
28E7-6	13120	14720	0.89
28F7-1	10040	9240	1.09
28F7-2	14240	11800	1.21
28F7-3	31080	29840	1.04
28F7-4	17720	17560	1.01
28F7-5	15520	12560	1.24
28F7-6	24240	22000	1.10
28G7-1	28000	11800	2.37
28G7-2	15240	14440	1.06
28G7-3	12720	11200	1.14
28G7-4	8440	7420	1.14
28G7-5	14160	12840	1.10
28G7-6	18480	17600	1.05
28B8-1	15080	12680	1.19
28B8-2	15400	13040	1.18
28B8-3	35560	32800	1.08
28B8-4	6900	7460	0.92
28B8-5	12760	12360	1.03
28B8-6	10000	11740	0.85
30E8-1	15920	15120	1.05
30E8-2	6980	6380	1.09
30E8-3	14920	13000	1.15
30E8-4	19320	19240	1.00
30E8-5	10620	10520	1.01
30E8-6	16860	16300	1.03
30F8-1	13220	13920	0.95
30F8-2	9580	9240	1.04
30F8-3	12490	12000	1.04
30F8-4	13820	13700	1.01
30F8-5	15200	14320	1.06
30F8-6	17600	8500	2.07
30G8-1	10640	6160	1.73
30G8-2	4400	3600	1.22
30G8-3	6200	4400	1.41
30G8-4	9100	7100	1.28
30G8-5	4000	2700	1.48
30G8-6	6900	6300	1.10

Clone ID	HC (FAM) 1 µL DNA final copies	COSMC (VIC) 1 µLDNA final copies	Target-reference ratio (HC copy #)
28F6-1	8960	9520	0.94
28F6-2	17360	16680	1.04
28F6-3	7540	7680	0.98
28F6-4	17720	15600	1.14
28F6-5	28160	28480	0.99
28F6-6	13640	14880	0.92
28E7-1	13000	12640	1.03
28E7-2	15680	15200	1.03
28E7-3	7920	7800	1.02
28E7-4	17160	17760	0.97
28E7-5	16320	17640	0.93
28E7-6	15360	19200	0.80
28F7-1	9800	10600	0.92
28F7-2	12360	12680	0.97
28F7-3	30040	31000	0.97
28F7-4	16120	17920	0.90
28F7-5	14440	15360	0.94
28F7-6	26640	27040	0.99
28G7-1	26520	13960	1.90
28G7-2	13760	14640	0.94
28G7-3	11080	11640	0.95
28G7-4	10080	10480	0.96
28G7-5	13280	12960	1.02
28G7-6	18960	18720	1.01
28B8-1	19440	21280	0.91
28B8-2	12800	16400	0.78
28B8-3	44000	43600	1.01
28B8-4	8140	6980	1.17
28B8-5	13080	12400	1.05
28B8-6	10840	13080	0.83
30E8-1	18960	17000	1.12
30E8-2	6480	7040	0.92
30E8-3	11160	11160	1.00
30E8-4	18200	18120	1.00
30E8-5	7680	7620	1.01
30E8-6	17250	16900	1.02
30F8-1	12880	11840	1.09
30F8-2	11380	10840	1.05
30F8-3	13970	13800	1.01
30F8-4	13520	13260	1.02
30F8-5	13280	14880	0.89
30F8-6	15800	15500	1.02
30G8-1	8880	9760	0.91
30G8-2	3800	6000	0.63
30G8-3	6200	7000	0.89
30G8-4	7000	9100	0.77
30G8-5	3400	4300	0.79
30G8-6	7400	7300	1.01
