# Supplementary material for: Synthetic promoters developed through a site-specific integration system enhance antibody expression in fed-batch culture
Source: Eng Life Sci. 2026 Jul 24;26(7):e005. doi: 10.48130/els-0026-0004 (PMC13599642; doi:10.48130/els-0026-0004)
Supplement: Supplementary file 1 [file els-2026-26-7-0004-S1.zip › 10.48130_els-0026-0004-Suppl-TableS11.pdf]

Table S11: Specific productivity and normalized mRNA level (D0-D4) of representative clonally-derived cell lines .

Clone ID	Specific productivity (pg/cell/day)	Normalized mRNA level (LC)	Normalized mRNA level (HC)
28F6-2	6.16	1.00	1.01
28E7-2	3.65	0.76	0.52
28F7-2	3.06	0.66	0.50
28G7-2	5.30	0.96	0.99
28B8-2	4.99	0.95	0.91
30E8-2	5.47	1.13	0.91
30F8-2	5.04	1.36	0.94
30G8-2	4.61	0.86	1.09
CMV-1	4.46	1.07	1.41
