# Supplementary material for: Synthetic promoters developed through a site-specific integration system enhance antibody expression in fed-batch culture
Source: Eng Life Sci. 2026 Jul 24;26(7):e005. doi: 10.48130/els-0026-0004 (PMC13599642; doi:10.48130/els-0026-0004)
Supplement: Supplementary file 1 [file els-2026-26-7-0004-S1.zip › 10.48130_els-0026-0004-Suppl-TableS12.pdf]

Table S12: Estimate of correct integration ratio of bsAb genes cassette before PCR test.

Promoters combination	# of monoclonal cells sorted by FACS	# of survived monoclonal cells	# of cells with FITC- & titer > 3 µg/µL	% of correct integration
C1	192	14	1	7.1
C2	192	35	27	77
