# Supplementary material for: Synthetic promoters developed through a site-specific integration system enhance antibody expression in fed-batch culture
Source: Eng Life Sci. 2026 Jul 24;26(7):e005. doi: 10.48130/els-0026-0004 (PMC13599642; doi:10.48130/els-0026-0004)
Supplement: Supplementary file 1 [file els-2026-26-7-0004-S1.zip › 10.48130_els-0026-0004-Suppl-TableS13.pdf]

Table S13: Copy number of four genes of bsAb in different monoclonal cells. Ratio between 0.5 to 1.5 is considered as single copy.

Clone ID	LC1 (FAM) 1 μL DNA final copies	COSMC (VIC) 1 μL DNA final copies	Target-reference ratio (LC copy #)
C1-1	5920	5720	1.03
C2-1	2400	2640	0.91
C2-2	6160	5320	1.16
C2-3	15600	16000	0.98

Clone ID	HC1 (FAM) 1 μL DNA final copies	COSMC (VIC) 1 μL DNA final copies	Target-reference ratio (LC copy #)
C1-1	4480	3880	1.15
C2-1	2740	2820	0.97
C2-2	5240	5360	0.98
C2-3	15700	16800	0.93

Clone ID	LC2 (FAM) 1 μL DNA final copies	COSMC (VIC) 1 μL DNA final copies	Target-reference ratio (LC copy #)
C1-1	5560	5920	0.94
C2-1	2860	3320	0.86
C2-2	5560	5560	1.00
C2-3	17400	15900	1.09

Clone ID	HC2 (FAM) 1 μL DNA final copies	COSMC (VIC) 1 μL DNA final copies	Target-reference ratio (LC copy #)
C1-1	5440	5320	1.02
C2-1	2640	2520	1.05
C2-2	6240	5520	1.13
C2-3	16700	15600	1.07
