# Supplementary material for: Synthetic promoters developed through a site-specific integration system enhance antibody expression in fed-batch culture
Source: Eng Life Sci. 2026 Jul 24;26(7):e005. doi: 10.48130/els-0026-0004 (PMC13599642; doi:10.48130/els-0026-0004)
Supplement: Supplementary file 1 [file els-2026-26-7-0004-S1.zip › 10.48130_els-0026-0004-Suppl-TableS14.pdf]

Table S14: Non-secreted mCherry fluorescence level (normalized) and secreted alkaline phosphatase (SEAP) signal [1]. Top 7 TFREs were selected based on reported data.

	SEAP transient	mCherry in transient	mCherry in SSI
NFκB	63	2.66	210.33
NFκB-p65	54	2.48	188.68
GABPβ	46	1.27	52.35
DMP1	38	1.43	29.42
AhR/ARNT	23	1.20	45.00
USF1	18	1.05	50.47
STAT3	17.5	0.57	1.46

Supplemental References:

[1] Johari, Y. B., Brown, A. J., Alves, C. S., Zhou, Y., Wright, C. M., Estes, S. D., Kshirsagar, R., & James, D. C. (2019). CHO genome mining for synthetic promoter design. *Journal of biotechnology*, *294*, 1–13.
