# Supplementary material for: Synthetic promoters developed through a site-specific integration system enhance antibody expression in fed-batch culture
Source: Eng Life Sci. 2026 Jul 24;26(7):e005. doi: 10.48130/els-0026-0004 (PMC13599642; doi:10.48130/els-0026-0004)
Supplement: Supplementary file 1 [file els-2026-26-7-0004-S1.zip › 10.48130_els-0026-0004-Suppl-TableS15.pdf]

Table S15. LC and HC gene expression were compared between synthetic promoter and CMV promoter using student’s t test. P-values were calculated.

	LC gene	HC gene
28F6	0.1412	0.0254
28E7	0.0011	0.0004
28F7	0.0002	0.0004
28G7	0.0121	0.0086
28B8	0.1134	0.0048
30E8	0.1601	0.0089
30F8	0.0009	0.0076
30G8	0.0012	0.0169
