# Supplementary material for: Synthetic promoters developed through a site-specific integration system enhance antibody expression in fed-batch culture
Source: Eng Life Sci. 2026 Jul 24;26(7):e005. doi: 10.48130/els-0026-0004 (PMC13599642; doi:10.48130/els-0026-0004)
Supplement: Supplementary file 1 [file els-2026-26-7-0004-S1.zip › 10.48130_els-0026-0004-Suppl-TableS2.pdf]

Table S2: Transcription factor regulatory element sequences

Transcription factor regulatory element	sequence
Activator protein 2 (AP-2)	TTGCCTGGGGCTAT
Activator protein 1 (AP1)	TGAGTCA
Amino acid RE (AARE)	ATTGCATCA
Antioxidant RE (ARE)	ATGACACAGCAAT
Aryl hydrocarbon receptor and nuclear translocator heterodimer (AhR/ARNT)	GTTGCGTGCGAA
Calcium-response factor (CaRF)	AGAACGAGGCA
cAMP RE (CRE)	TGACGTCA
CCAAT-enhancer binding protein epsilon (C/EBPε)	ATTGCGCAAT
Cyclin D-interacting Myb-like protein (DMP1)	GACCCGGATGTAG
D-box	ATTATGTAAC
Delta-lactoferrin (ΔLf)	GGCACTTGC
Dioxin RE (DRE)	GCTTGCGTGAGAAG
E2F transcription factor 3 (E2F3)	GCTCGGCGCCAAAC
Early growth response protein 1 (EGR-1)	ATGCGTGGGCGT
Endoplasmic reticulum stress RE (ERSE)	CCAATGGCCAGCCTCCACG
Enhancer box (E-box)	CACGTG
Erythroid Krueppel-like factor (EKLF)	CAGGGAGGGTG
Estrogen-related receptor alpha (ERRα)	TCCAAGGTCACA
ETS binding site 1 (EBS1)	ACCGGAAGT
GA-binding protein beta (GABPβ)	CCCCGGAAGTGAC
GC-box	GGGGCGGGG
GLI family zinc finger protein 2 (GLI2)	GACCACCCAAG
Glial cells missing homolog 1 (GCM1)	AAACCCGCATAT
Gut-enriched Krueppel-like factor (GKLF)	ATCACAGGATT
Hypoxia RE (HRE)	GTACGTGC
Myeloid zinc finger 1 (MZF1)	TGGTGGGGAGGGG
Nuclear factor 1 RE (NF1-RE)	TTGGCTATATGCCAA
Nuclear factor I-C (NFIC)	AGCCAA
Nuclear factor kappaB (NFκB)	TGGGACTTTCCA
Nuclear factor kappaB p65 (NFκB-p65)	TTGGGGATTTCCCA
Oocyte specific homeobox 2 (Obox2)	TAGTTAATCCCCCT
Paired box protein Pax-3 (Pax3)	TCGTCACGCTTCA
Paired box protein Pax-9 (Pax9)	GTCACGCATGACTGC
Rat Olf-1/EBF-associated Zn finger protein (ROAZ)	GGCACCCAAGGGTGA
Signal transducer/activator of transcription 3 (STAT3)	ATTTCCCGGAAATG
Sterol regulatory element binding protein 1 & 2 (SREBP)	AATCACCCCACTGC
Stimulating protein 1 (Sp1)	AGGGGCGGGGT
T-cell leukemia homeobox 1 (TLX1)	CGGTAATTGG
Upstream stimulating factor 1 (USF1)	GGGTCACGTGG
Viral Myb protein (v-Myb)	TTTAACGGCAA
v-rel avian reticuloendotheliosis viral oncogene homolog domain (REL)	GGAAAGTCCC
v-rel avian reticuloendotheliosis viral oncogene homolog domain A (RELA)	GGAAAGTACC
Wilms tumor suppressor (WT1)	TGCGTGGGAGTAG
Zinc finger BED-type containing 1 (ZBED1)	TGTCGCGACA
