# Supplementary material for: Synthetic promoters developed through a site-specific integration system enhance antibody expression in fed-batch culture
Source: Eng Life Sci. 2026 Jul 24;26(7):e005. doi: 10.48130/els-0026-0004 (PMC13599642; doi:10.48130/els-0026-0004)
Supplement: Supplementary file 1 [file els-2026-26-7-0004-S1.zip › 10.48130_els-0026-0004-Suppl-TableS3.pdf]

Table S3: Heterotypic promoters were composed of TFREs with corresponding copy number from different pools. The dark, medium and light shading corresponded to strong, moderate and weak TFREs respectively.

Pool A	copy number
NFκB	8
REL	8
GABPβ	2
SP1	2
DMP1	4
AhR/ARNT	4
**Total TFRE copies**	**28**

Pool B	copy number
NFκB	8
REL	8
GABPβ	2
SP1	2
DMP1	4
AhR/ARNT	4
CaRF	2
**Total TFRE copies**	**30**

Pool C	copy number
AP1	12
REL	8
NFκB	8
ERSE	8
DRE	4
E-box	8
GC-box	2
ARE	8
**Total TFRE copies**	**58**

Pool D	copy number
AP1	12
REL	8
NFκB	8
ERSE	8
DRE	4
E-box	4
GC-box	2
ARE	4
EBS1	2
GABPβ	2
SP1	2
CRE	2
**Total TFRE copies**	**58**
