# Supplementary material for: Synthetic promoters developed through a site-specific integration system enhance antibody expression in fed-batch culture
Source: Eng Life Sci. 2026 Jul 24;26(7):e005. doi: 10.48130/els-0026-0004 (PMC13599642; doi:10.48130/els-0026-0004)
Supplement: Supplementary file 1 [file els-2026-26-7-0004-S1.zip › 10.48130_els-0026-0004-Suppl-TableS4.pdf]

Table S4: Sequences of all genes of interest (GOIs), promoters, poly (A) tail, *lox* sites, primers and probes used in this study.

Genes of interest	sequence
mCherry	ATGGTGAGCAAGGGCGAGGAGGATAACATGGCCATCATCAAGGAGTTCATGCGCTTCAAGGT<br>GCACATGGAGGGCTCCGTGAACGGCCACGAGTTCGAGATCGAGGGCGAGGGCGAGGGCCG<br>CCCCTACGAGGGCACCCAGACCGCCAAGCTGAAGGTGACCAAGGGTGGCCCCCTGCCCTTC<br>GCCTGGGACATCCTGTCCCCTCAGTTCATGTACGGCTCCAAGGCCTACGTGAAGCACCCCGC<br>CGACATCCCCGACTACTTGAAGCTGTCCTTCCCCGAGGGCTTCAAGTGGGAGCGCGTGATGA<br>ACTTCGAGGACGGCGGCGTGGTGACCGTGACCCAGGACTCCTCCCTGCAGGACGGCGAGTT<br>CATCTACAAGGTGAAGCTGCGCGGCACCAACTTCCCCTCCGACGGCCCCGTAATGCAGAAGA<br>AGACCATGGGCTGGGAGGCCTCCTCCGAGCGGATGTACCCCGAGGACGGCGCCCTGAAGGG<br>CGAGATCAAGCAGAGGCTGAAGCTGAAGGACGGCGGCCACTACGACGCTGAGGTCAAGACC<br>ACCTACAAGGCCAAGAAGCCCGTGCAGCTGCCCGGCGCCTACAACGTCAACATCAAGTTGGA<br>CATCACCTCCCACAACGAGGACTACACCATCGTGGAACAGTACGAACGCGCCGAGGGCCGCC<br>ACTCCACCGGCGGCATGGACGAGCTGTACAAGTAG
PuroR	ATGACCGAGTACAAGCCCACGGTGCGCCTCGCCACCCGCGACGACGTCCCCAGGGCCGTAC<br>GCACCCTCGCCGCCGCGTTCGCCGACTACCCCGCCACGCGCCACACCGTCGATCCGGACCG<br>CCACATCGAGCGGGTCACCGAGCTGCAAGAACTCTTCCTCACGCGCGTCGGGCTCGACATCG<br>GCAAGGTGTGGGTCGCGGACGACGGCGCCGCGGTGGCGGTCTGGACCACGCCGGAGAGCG<br>TCGAAGCGGGGGCGGTGTTCGCCGAGATCGGCCCGCGCATGGCCGAGTTGAGCGGTTCCCG<br>GCTGGCCGCGCAGCAACAGATGGAAGGCCTCCTGGCGCCGCACCGGCCCAAGGAGCCCGC<br>GTGGTTCCTGGCCACCGTCGGCGTCTCGCCCGACCACCAGGGCAAGGGTCTGGGCAGCGC<br>CGTCGTGCTCCCCGGAGTGGAGGCGGCCGAGCGCGCCGGGGTGCCCGCCTTCCTGGAGAC<br>CTCCGCGCCCCGCAACCTCCCCTTCTACGAGCGGCTCGGCTTCACCGTCACCGCCGACGTC<br>GAGGTGCCCGAAGGACCGCGCACCTGGTGCATGACCCGCAAGCCCGGTGCCTGA
msAb LC	ATGTCTGTGCCTACACAGGTTCTGGGACTGCTGCTGCTGTGGCTGACAGACGCCAGATGCGA<br>CATCGTGATGACACAGAGCCCTAGCAGCCTGGCTGTGTCTGTGGGAGAGAGAGTGACCATCA<br>GCTGCAAGAGCAGCCAGAGCCTGCTGTACTCCACCAACCAGAAGAACTACCTGGCCTGGTAT<br>CAGCAGAAGCCCGGCCAGTCTCCTAAGCTGCTGATCTACTGGGCCAGCACCAGAGAAAGCGG<br>CGTGCCAGATAGATTCAGCGGCAGCGGCTCTGGAACCGACTTCACCCTGACAATCAGCTCCC<br>TGCAGGCCGAGGATGTGGCCGTGTACTACTGCCAGCAGTACTACAGCTACCTGAGAACCTTCG<br>GCGGAGGCACCAAGCTGGAAATCAAGAGAACAGTGGCCGCTCCTAGCGTGTTCATCTTCCCA<br>CCATCTGACGAGCAGCTGAAGTCTGGCACAGCCAGCGTTGTGTGCCTGCTGAACAACTTCTA<br>CCCCAGAGAAGCCAAGGTGCAGTGGAAGGTGGACAACGCTCTGCAGTCCGGCAACAGCCAA<br>GAGAGCGTGACAGAGCAGGACAGCAAGGACTCCACCTACAGCCTGAGCAGCACACTGACAC<br>TGAGCAAGGCCGACTACGAAAAGCACAAGGTGTACGCCTGCGAAGTGACCCACCAGGGACTG<br>TCTAGCCCTGTGACCAAGAGCTTCAACAGAGGCGAGTGCTGA
msAb HC	ATGGAATGGTCCTGGGTGTTCCTGTTCTTCCTGAGCGTGACCACAGGCGTGCACTCTGAGGT<br>GCAGCTGGTTGAATCTGGCGGAGGACTGGTTCAGCCTGGCGGATCTCTGAGACTGTCTTGTG<br>CCGCCAGCGGCTTCACCTTCAGCAGCTACGCTATGAGCTGGGTCCGACAGGCTCCTGGCAAA<br>GGACTTGAGTGGGTCGCCTACATCAGCAGCGGCGGCAGCACATATTACCCCGACTCTGTGAA<br>GGGCAGATTCACCATCTCCAGAGACAACGCCAAGAACAGCCTGTACCTGCAGATGAACTCCCT<br>GAGAGCCGAGGACACCGCCGTGTACTACTGTGCCAGAGAAGGCTACGGCACCTACGGCGATT<br>ATTGGGGCCAGGGCACAACCCTGACAGTGTCTAGCGCTTCCACAAAGGGCCCCAGCGTTTTC<br>CCACTGGCTCCTAGCAGCAAGAGCACAAGCGGAGGAACAGCTGCTCTGGGCTGTCTGGTCAA<br>GGACTACTTCCCTGAGCCTGTGACCGTGTCCTGGAACTCTGGCGCTCTGACTTCTGGCGTGC<br>ACACATTCCCTGCCGTGCTGCAATCTAGCGGCCTGTACTCTCTGAGCAGCGTGGTCACAGTGC<br>CAAGCTCTAGCCTGGGCACCCAGACCTACATCTGCAATGTGAACCACAAGCCTAGCAACACCA<br>AGGTGGACAAGAAGGTGGAACCCAAGAGCTGCGACAAGACCCACACCTGTCCTCCATGTCCT<br>GCTCCAGAACTGCTCGGCGGACCTTCCGTGTTTCTGTTCCCTCCAAAGCCTAAGGACACCCT<br>GATGATCAGCAGAACCCCTGAAGTGACCTGCGTGGTGGTGGATGTGTCTCACGAGGACCCCG<br>AAGTGAAGTTCAATTGGTACGTGGACGGCGTGGAAGTGCACAACGCTAAGACCAAGCCTAGA<br>GAGGAACAGTACAACAGCACCTATAGAGTGGTGTCCGTGCTGACAGTGCTGCACCAGGATTG<br>GCTGAACGGCAAAGAGTACAAGTGCAAGGTGTCCAACAAGGCCCTGCCTGCTCCTATCGAAA<br>AGACCATCAGCAAGGCCAAGGGCCAGCCTAGGGAACCCCAGGTTTACACACTGCCACCAAGC<br>AGGGACGAGCTGACCAAGAATCAGGTGTCCCTGACCTGCCTCGTGAAGGGCTTCTACCCTTC<br>CGATATCGCCGTGGAATGGGAGAGCAACGGCCAGCCTGAGAACAACTACAAGACAACCCCTC<br>CTGTGCTGGACAGCGACGGCTCATTCTTCCTGTACAGCAAGCTGACCGTGGACAAGTCCAGA<br>TGGCAGCAGGGCAACGTGTTCAGCTGCAGCGTGATGCACGAGGCCCTGCACAACCACTACAC<br>CCAGAAGTCCCTGTCTCTGAGCCCCGGCAAATGA
bsAb LC1	ATGGTCCTGGGTGTTCCTGTTCTTCCTGTCTCTGACCGGGGTGCACGCCGACATCCAGATGAC<br>ACAGTCCCCATCTTCTCTGTCCGCCTCTGTGGGCGACAGAGTGACCATCACCTGTAGAGCCA<br>GCCAGGACATCTCCAACTACCTGAACTGGTATCAGCAGAAGCCCGACGGCACCATCAAGCTG<br>CTGATCTACTACACCTCCAGACTGCACTCTGGCGTGCCCTCCAGATTTTCTGGCTCTGGATCT<br>GGCACCGACTACACCTTCACCATCTCTAGCCTGCAGCCTGAGGATATCGCCACCTACTACTGC

	CAGCAGGGCAATACCCTGCCTTGGACCTTTGGCGGCGGAACAAAGGTGGAAATCAAAGGCGG<br>CGGAGGAAGCTCTGGCGCCCCTAGATTTCTGACCAGACCTAAGGCTTCCGTGGTGTCCGTGG<br>GCAAAGACGCTACCCTGTCTTGTCAGATCGTGGGCAACCCATTTCCTCAGGTGTCCTGGGAGA<br>AAGACAAGCAGCCTGTGACAGCCGGCGTGCGGTTTAGATTGGCTCAGGATGGCGACCTGTAC<br>CGGCTGAAGATCCTCGACCTGCAGCTGTCTGATTCCGGCCAGTACGTGTGCAGAGCTAGAAAT<br>GCCCACGGCGAGGCTTTTGCTTGTCTGGGACTGCAAGTGGATGCCGAGGCTTGA
bsAb HC1	ATGGAATGGTCCTGGGTGTTCCTGTTCTTCCTGTCTCTGACTGGGGTGCACGCTGAAGTGCAG<br>TTGGTTCAGAGCGGAGCCGAAGTGAAGAAACCTGGCGCTTCTGTGAAGGTGTCCTGCAAGGC<br>TTCTGGCTACACCCTGACCAGCTACTGGATCACCTGGGTCCGACAAGCTCCTGGACAGGGAC<br>TTGAGTGGATGGGCGATATCTATCCTGGCAGCGGCCGGACCAACTACAACGAGATGTTCAAGA<br>ACAGAGTGACCATGACCGTGGACACCTCCACCAGCACCGCTTACATGGAACTGTCCAGCCTG<br>AGATCTGAGGACACCGCCGTGTACTACTGCGCTAGAAGGCACTATGGCACCGGCTACGAGTCT<br>TTCGATGTGTGGGGACAGGGCACCACCGTGACAGTTTCTAGCGGAGGCGGAGGATCTGGCAT<br>CCCTCCTAAGATCGAGTGCCTGCCTATCGACATCTCCATCGACGAAGGCAAGGTGCTGACCGT<br>GGCTTCTGCTTTTACTGGCGAGCCCACACCTGAAGTGACCTGGTCTACCGGCGGCAGAAAGA<br>TCCACTCTCAAGAGCAGGGCAGATTCCACATCGAGAACACCGACGACTCCACCACACTGACC<br>ATCAAGGACGTGCAGAAACAGGACGGCGGCCTGTACACTCTGACCCTGAGAAACGAGTTCGG<br>CTCTGACTCCGCCACCGTGAACATCCACATCCGGTCCATCGACAAGACCCACACATGCCCTCC<br>ATGTCCTGCTCCAGAAGCTGCTGGCGGACCCTCTGTGTTTCTGTTCCCTCCAAAGCCTAAGGA<br>CACCCTGATGATCTCTCGGACTCCCGAAGTGACATGCGTGGTGGTGGATGTGTCTCATGAGGA<br>CCCAGAAGTGAAGTTCAATTGGTACGTGGACGGCGTGGAAGTGCACAACGCCAAGACCAAGC<br>CTAGAGAGGAACAGTACAACTCCACCTACAGAGTGGTGTCCGTGCTGACAGTGCTGCACCAG<br>GATTGGCTGAACGGCAAAGAGTACAAGTGCAAGGTGTCCAACAAGGCCCTGCCTGCTCCTAT<br>CGAAAAGACCATCTCCAAGGCTAAGGGCCAGCCTCGGGAACCTCAGGTTTACACACTGCCTC<br>CATGCCGGGACGAGCTGACCAAAAATCAGGTGTCCCTGTGGTGCCTGGTCAAGGGCTTCTAC<br>CCTTCCGATATCGCCGTGGAATGGGAGTCTAACGGCCAGCCAGAGAACAACTACAAGACCACA<br>CCTCCTGTGCTGGACTCCGACGGCTCATTCTTTCTGTACTCCAAGCTGACTGTGGACAAGTCC<br>AGATGGCAGCAGGGCAACGTGTTCTCCTGCTCTGTGATGCACGAGGCCCTGCACAACCACTA<br>CACACAGAAATCCCTGAGCCTGTCTCCTGGCAAATGA
bsAb LC2	ATGGTCCTGGGTGTTCCTGTTCTTCCTGTCTCTGACCGGGGTGCACGCCGATATCCAGCTGAC<br>ACAGTCCCCATCTTCTCTGTCTGCCTCTGTGGGCGACAGAGTGACCATCACCTGTAGAGCCAG<br>CCAGGACATCTCTAGCGCCCTCGTGTGGTATCAGCAGAAGCCTGGAAAGGCCCCTAAGCTGC<br>TGATCTACGACGCCTCCTCTCTGGAATCTGGCGTGCCATCCAGATTCTCCGGCTCTGAGTCTG<br>GCACCGACTTCACCCTGACAATCTCTAGCCTGCAGCCTGAGGACTTCGCCACCTACTACTGCC<br>AGCAGTTCAACAGCTACCCTCTGACCTTTGGCGGAGGCACCAAGGTGGAAATCAAGAGAACC<br>GTGGCCGCTCCTTCCGTGTTCATCTTCCCACCATCTGACGAGCAGCTGAAGTCCGGCACAGC<br>TTCTGTCGTGTGCCTGCTGAACAACTTCTACCCTCGGGAAGCCAAGGTGCAGTGGAAGGTGG<br>ACAATGCCCTGCAGTCCGGCAACTCCCAAGAGTCTGTGACCGAGCAGGACTCCAAGGACTCT<br>ACCTACAGCCTGTCCTCCACACTGACCCTGTCTAAGGCCGACTACGAGAAGCACAAGGTGTAC<br>GCCTGTGAAGTGACCCACCAGGGACTGTCTAGCCCCGTGACCAAGTCTTTCAACAGAGGCGA<br>GTGCTGA
bsAb HC2	ATGGTCCTGGGTGTTCCTGTTCTTCCTGTCTCTGACTGGGGTGCACGCTCAGGTGCAGTTGG<br>TTGAATCTGGTGGCGGAGTGGTGCAGCCTGGCAGATCTCTGAGACTGTCTTGTGCCGCCTCC<br>GGCTTCACCTTCTCTACCTATGGCATGCACTGGGTCCGACAGGCCCCTGGAAAAGGACTGGA<br>ATGGGTTGCCGTGATCTGGGACGACGGCTCCTACAAGTACTACGGCGACTCCGTGAAGGGCA<br>GATTCACCATCTCTCGGGACAACTCCAAGAACACCCTGTACCTGCAGATGAACTCCCTGAGAG<br>CCGAGGACACCGCCGTGTACTATTGTGCCAGAGATGGCATCACCATGGTCCGAGGCGTGATG<br>AAGGACTACTTCGATTATTGGGGCCAGGGCACCCTGGTCACCGTGTCCTCTGCTTCTACAAAG<br>GGCCCCTCTGTGTTCCCTCTGGCTCCTTCCTCTAAATCCACCTCTGGCGGAACCGCTGCTCTG<br>GGCTGTCTGGTCAAGGATTACTTCCCTGAGCCTGTGACCGTGTCTTGGAACTCTGGTGCTCTG<br>ACATCCGGCGTGCACACCTTTCCAGCTGTGCTGCAGTCCTCTGGCCTGTACTCTCTGTCCTCT<br>GTCGTGACCGTGCCTTCTAGCTCTCTGGGCACCCAGACCTACATCTGCAACGTGAACCACAAG<br>CCTTCCAACACCAAGGTGGACAAGAAGGTGGAACCCAAGTCCTGCGACAAGACCCACACCTG<br>TCCTCCATGTCCTGCTCCAGAAGCTGCTGGCGGACCCTCCGTTTTCCTGTTTCCACCTAAGCC<br>TAAGGACACCCTGATGATCTCTCGGACCCCTGAAGTGACCTGCGTGGTGGTGGATGTGTCTCA<br>CGAGGATCCCGAAGTGAAGTTCAATTGGTACGTGGACGGCGTGGAAGTGCACAACGCCAAGA<br>CCAAGCCTAGAGAGGAACAGTACAACTCCACCTACAGAGTGGTGTCCGTGCTGACCGTGCTG<br>CACCAGGATTGGCTGAACGGCAAAGAGTACAAGTGCAAGGTGTCCAACAAGGCCCTGCCTGC<br>TCCTATCGAAAAGACCATCTCCAAGGCTAAGGGCCAGCCTCGGGAACCTCAAGTCTGTACACT<br>GCCTCCTAGCCGGGACGAGCTGACCAAAAATCAGGTGTCCCTGTCCTGCGCTGTGAAGGGCT<br>TCTACCCTTCCGATATCGCCGTGGAATGGGAGTCCAATGGCCAGCCTGAGAACAACTACAAGA<br>CAACCCCTCCTGTGCTGGACTCCGACGGCTCATTCTTCCTGGTGTCCAAGCTGACAGTGGAC<br>AAGTCCAGATGGCAGCAGGGCAACGTGTTCTCCTGCTCTGTGATGCACGAGGCCCTGCACAA<br>CCACTACACCCAGAAGAGTCTGTCTCTGTCCCCTGGCAAATGA

Promoters and poly(A) tail	sequence
Proprietary CMV [2]	TGAGGCGCGCCTACTGAGTCATTAGGGACTTTCCAATGGGTTTTGCCCAGTACATAAGGTCAAT<br>AGGGGTGAATCAACAGGAAAGTCCCATTGGAGCCAAGTACACTGAGTCAATAGGGACTTTCCA<br>TTGGGTTTTGCCCAGTACAAAAGGTCAATAGGGGGTGAGTCAATGGGTTTTTCCCATTATTGGC<br>ACGTACATAAGGTCAATAGGGGTGAGTCATTGGGTTTTTCCAGCCAATTTAATTAAAACGCCATG<br>TACTTTCCCACCATTGACGTCAATGGGCTATTGAAACTAATGCAACGTGACCTTTAAACGGTACT<br>TTCCCATAGCTGATTAATGGGAAAGTACCGTTCTCGAGCCAATACACGTCAATGGGAAGTGAAA<br>GGGCAGCCAAAACGTAACACCGCCCCGGTTTTCCCCTGGAAATTCCATATTGGCACGCATTCT<br>ATTGGCTGAGCTGCGTTCTACGTGGGTATAAGAGGCGCGACCAGCGTCGGTACCGTCGCAGT<br>CTTCGGTCTGACCACCGTAGAACGCAGCCTCAGGACCTCCATAGAAGACACCGGGACCGATC<br>CAGCCTCCGCGGCCGGGAACGGTGCATTGGAACGCGGATTCCCCGTGCCAAGAGTGACGTA<br>AGTACCGCCTATAGAGTCTATAGGCCCACCCCCTTGGCTTCTTATGCATGCTATACTGTTTTTGG<br>CTTGGGGTCTATACACCCCCGCTTCCTCATGTTATAGGTGATGGTATAGCTTAGCCTATAGGTGT<br>GGGTTATTGACCATTATTGACCACTCCCCTATTGGTGACGATACTTTCCATTACTAATCCATAACA<br>TGGCTCTTTGCCACAACTCTCTTTATTGGCTATATGCCAATACACTGTCCTTCAGAGACTGACAC<br>GGACTCTGTATTTTTACAGGATGGGGTCTCATTTATTATTTACAAATTCACATATACAACACCACC<br>GTCCCCAGTGCCCGCAGTTTTTATTAAACATAACGTGGGATCTCCACGCGAATCTCGGGTACGT<br>GTTCCGGACATGGGCTCTTCTCCGGTAGCGGCGGAGCTTCTACATCCGAGCCCTGCTCCCAT<br>GCCTCCAGCGACTCATGGTCGCTCGGCAGCTCCTTGCTCCTAACAGTGGAGGCCAGACTTAG<br>GCACAGCACGATGCCCACCACCACCAGTGTGCCGCACAAGGCCGTGGCGGTAGGGTATGTG<br>TCTGAAAATGAGCTCGGGGAGCGGGCTTGCACCGCTGACGCATTTGGAAGACTTAAGGCAGC<br>GGCAGAAGAAGATGCAGGCAGCTGAGTTGTTGTGTTCTGATAAGAGTCAGAGGTAACTCCCGT<br>TGCGGTGCTGTTAACGGTGGAGGGCAGTGTAGTCTGAGCAGTACTCGTTGCTGCCGCGCGCG<br>CCACCAGACATAATAGCTGACAGACTAACAGACTGTTCCTTTCCATGGGTCTTTTCTGCAGTCA<br>CCGTCCTTGACACG
100 RPU.2 [3]	TGGGACTTTCCACCTTAGATGACACAGCAATCAGATTTGCTTGCGTGAGAAGATATAGGATGAC<br>ACAGCAATCTAGACTGGGACTTTCCACTGATATTTTGCGCAATTGACCTAATGACACAGCAATAG<br>TATGTGGGGCGGGGATCTAACTGGGACTTTCCAAAGGTCTTACCGGAAGTTGTTAGAATGACA<br>CAGCAATGGATTCATATCCTGGGACTTTCCAGTATACTGCTTGCGTGAGAAG<br>ATGATCATGGGACTTTCCA
CMV core promoter	AGGTCTATATAAGCAGAGCTCGTTTAGTGAACCGTCAGATCGCCTAGATACGCCATCCACGCT<br>GTTTTGACCTCCATAGAAGAC
EF1α	GGCTCCGGTGCCCGTCAGTGGGCAGAGCGCACATCGCCCACAGTCCCCGAGAAGTTGGGG<br>GGAGGGGTCGGCAATTGAACCGGTGCCTAGAGAAGGTGGCGCGGGGTAAACTGGGAAAGTG<br>ATGTCGTGTACTGGCTCCGCCTTTTTCCCGAGGGTGGGGGAGAACCGTATATAAGTGCAGTA<br>GTCGCCGTGAACGTTCTTTTTCGCAACGGGTTTGCCGCCAGAACACAGGTAAGTGCCGTGTG<br>TGGTTCCCGCGGGCCTGGCCTCTTTACGGGTTATGGCCCTTGCGTGCCTTGAATTACTTCCA<br>CCTGGCTCCAGTACGTGATTCTTGATCCCGAGCTGGAGCCAGGGGCGGGCCTTGCGCTTTAG<br>GAGCCCCTTCGCCTCGTGCTTGAGTTGAGGCCTGGCCTGGGCGCTGGGGCCGCCGCGTGC<br>GAATCTGGTGGCACCTTCGCGCCTGTCTCGCTGCTTTCGATAAGTCTCTAGCCATTTAAAATTT<br>TTGATGACCTGCTGCGACGCTTTTTTTCTGGCAAGATAGTCTTGTAAATGCGGGCCAGGATCT<br>GCACACTGGTATTTCGGTTTTTGGGGCCGCGGGCGGCGACGGGGCCCGTGCGTCCCAGCGC<br>ACATGTTCGGCGAGGCGGGGCCTGCGAGCGCGGCCACCGAGAATCGGACGGGGGTAGTCT<br>CAAGCTGGCCGGCCTGCTCTGGTGCCTGGCCTCGCGCCGCCGTGTATCGCCCCGCCCTGGG<br>CGGCAAGGCTGGCCCGGTCGGCACCAGTTGCGTGAGCGGAAAGATGGCCGCTTCCCGGCC<br>CTGCTCCAGGGGGCTCAAAATGGAGGACGCGGCGCTCGGGAGAGCGGGCGGGTGAGTCAC<br>CCACACAAAGGAAAGGGGCCTTTCCGTCCTCAGCCGTCGCTTCATGTGACTCCACGGAGTAC<br>CGGGCGCCGTCCAGGCACCTCGATTAGTTCTGGAGCTTTTGGAGTACGTCGTCTTTAGGTTG<br>GGGGGAGGGGTTTTATGCGATGGAGTTTCCCCACACTGAGTGGGTGGAGACTGAAGTTAGG<br>CCAGCTTGGCACTTGATGTAATTCTCCTTGGAATTTGCCCTTTTTGAGTTTGGATCTTGGTTCA<br>TTCTCAAGCCTCAGACAGTGGTTCAAAGTTTTTTTCTTCCATTTCAGGTGTCGTGA
SV40	GTGTGTCAGTTAGGGTGTGGAAAGTCCCCAGGCTCCCCAGCAGGCAGAAGTATGCAAAGCAT<br>GCATCTCAATTAGTCAGCAACCAGGTGTGGAAAGTCCCCAGGCTCCCCAGCAGGCAGAAGTAT<br>GCAAAGCATGCATCTCAATTAGTCAGCAACCATAGTCCCGCCCCTAACTCCGCCCATCCCGCC<br>CCTAACTCCGCCCAGTTCCGCCCATTCTCCGCCCCATGGCTGACTAATTTTTTTTATTTATGCAG<br>AGGCCGAGGCCGCCTCTGCCTCTGAGCTATTCCAGAAGTAGTGAGGAGGCTTTTTTGGAGGC<br>CTAGGCTTTTGCAAA

Enhancer sequence	Sequence (CMV core promoter not included)

28F6	CATAGGGGCGGGGTCATGGAAAGTCCCCATGACCCGGATGTAGCATCC<br>CCGGAAGTGACCATGGAAAGTCCCCATGGAAAGTCCCCATGGAAAGTC<br>CCCATGTTGCGTGCGAACATGACCCGGATGTAGCATGGAAAGTCCCCA<br>TGACCCGGATGTAGCATGTTGCGTGCGAACATTGGGACTTTCCACATGG<br>AAAGTCCCCATTGGGACTTTCCACATTGGGACTTTCCACATCCCCGGAA<br>GTGACCATGTTGCGTGCGAACATAGGGGCGGGGTCATTGGGACTTTCC<br>ACATGGAAAGTCCCCATGTTGCGTGCGAACATTGGGACTTTCCACATGA<br>CCCGGATGTAGCATTGGGACTTTCCACATGGAAAGTCCCCATTGGGACT<br>TTCCACATTGGGACTTTCCACAT
28E7	CATGTTGCGTGCGAACATTGGGACTTTCCACATGACCCGGATGTAGCAT<br>TGGGACTTTCCACATTGGGACTTTCCACATTGGGACTTTCCACATAGGG<br>GCGGGGTCATTGGGACTTTCCACATGGAAAGTCCCCATGTTGCGTGCGA<br>ACATGTTGCGTGCGAACATTGGGACTTTCCACATGGAAAGTCCCCATCC<br>CCGGAAGTGACCATGGAAAGTCCCCATGGAAAGTCCCCATGACCCGGA<br>TGTAGCATCCCCGGAAGTGACCATTGGGACTTTCCACATGGAAAGTCCC<br>CATGGAAAGTCCCCATTGGGACTTTCCACATGGAAAGTCCCCATAGGG<br>GCGGGGTCATGGAAAGTCCCCATGACCCGGATGTAGCATGTTGCGTGC<br>GAACATGACCCGGATGTAGCAT
28F7	CATGTTGCGTGCGAACATTGGGACTTTCCACATTGGGACTTTC<br>CACATTGGGACTTTCCACATGGAAAGTCCCCATGGAAAGTCCC<br>CATGTTGCGTGCGAACATAGGGGCGGGGTCATTGGGACTTTCC<br>ACATGACCCGGATGTAGCATCCCCGGAAGTGACCATGTTGCGT<br>GCGAACATCCCCGGAAGTGACCATTGGGACTTTCCACATAGGG<br>GCGGGGTCATGGAAAGTCCCCATTGGGACTTTCCACATGGAAA<br>GTCCCCATGACCCGGATGTAGCATGGAAAGTCCCCATGTTGCG<br>TGCGAACATGGAAAGTCCCCATGACCCGGATGTAGCATTGGGA<br>CTTTCCACATGGAAAGTCCCCATTGGGACTTTCCACATGGAAA<br>GTCCCCATGACCCGGATGTAGCAT
28G7	CATGGAAAGTCCCCATGACCCGGATGTAGCATAGGGGCGGGGT<br>CATGGAAAGTCCCCATTGGGACTTTCCACATAGGGGCGGGGTC<br>ATCCCCGGAAGTGACCATCCCCGGAAGTGACCATGGAAAGTC<br>CCCATGTTGCGTGCGAACATGACCCGGATGTAGCATGGAAAGT<br>CCCCATGGAAAGTCCCCATTGGGACTTTCCACATGGAAAGTCC<br>CCATTGGGACTTTCCACATGTTGCGTGCGAACATGGAAAGTCC<br>CCATGACCCGGATGTAGCATTGGGACTTTCCACATTGGGACTTT<br>CCACATGACCCGGATGTAGCATTGGGACTTTCCACATTGGGAC<br>TTTCCACATGTTGCGTGCGAACATGTTGCGTGCGAACATGGAA<br>AGTCCCCATTGGGACTTTCCACAT
28B8	CATCCCCGGAAGTGACCATGACCCGGATGTAGCATGGAAAGTCCCCAT<br>GTTGCGTGCGAACATGGAAAGTCCCCATAGGGGCGGGGTCATCCCCGG<br>AAGTGACCATGGAAAGTCCCCATGGAAAGTCCCCATTGGGACTTTCCA<br>CATGTTGCGTGCGAACATGGAAAGTCCCCATGACCCGGATGTAGCATT<br>GGGACTTTCCACATTGGGACTTTCCACATTGGGACTTTCCACATTGGGA<br>CTTTCCACATAGGGGCGGGGTCATTGGGACTTTCCACATGGAAAGTCCC<br>CATGACCCGGATGTAGCATGTTGCGTGCGAACATGTTGCGTGCGAACA<br>TGACCCGGATGTAGCATGGAAAGTCCCCATGGAAAGTCCCCATTGGGA<br>CTTTCCACATTGGGACTTTCCACAT
30E8	CATCCCCGGAAGTGACCATTGGGACTTTCCACATGTTGCGTGCGAACAT<br>GACCCGGATGTAGCATGGAAAGTCCCCATTGGGACTTTCCACATGTTGC<br>GTGCGAACATAGGGGCGGGGTCATGGAAAGTCCCCATGGAAAGTCCCC<br>ATGACCCGGATGTAGCATGACCCGGATGTAGCATGGAAAGTCCCCATG<br>TTGCGTGCGAACATTGGGACTTTCCACATGGAAAGTCCCCATGGAAAG<br>TCCCCATAGAACGAGGCACATTGGGACTTTCCACATGGAAAGTCCCCA<br>TGTTGCGTGCGAACATAGGGGCGGGGTCATTGGGACTTTCCACATCCCC<br>GGAAGTGACCATGGAAAGTCCCCATTGGGACTTTCCACATGACCCGGA<br>TGTAGCATTGGGACTTTCCACATAGAACGAGGCACATTGGGACTTTCCA<br>CAT
30F8	CATTGGGACTTTCCACATTGGGACTTTCCACATCCCCGGAAGTGACCAT<br>TGGGACTTTCCACATTGGGACTTTCCACATGGAAAGTCCCCATGACCCG<br>GATGTAGCATTGGGACTTTCCACATAGAACGAGGCACATGACCCGGAT<br>GTAGCATGACCCGGATGTAGCATGACCCGGATGTAGCATGGAAAGTCC<br>CCATGTTGCGTGCGAACATGGAAAGTCCCCATAGAACGAGGCACATGG<br>AAAGTCCCCATGTTGCGTGCGAACATCCCCGGAAGTGACCATGTTGCGT<br>GCGAACATGTTGCGTGCGAACATAGGGGCGGGGTCATTGGGACTTTCC<br>ACATGGAAAGTCCCCATGGAAAGTCCCCATAGGGGCGGGGTCATGGAA<br>AGTCCCCATTGGGACTTTCCACATGGAAAGTCCCCATTGGGACTTTCCA<br>CAT

30G8	CATGACCCGGATGTAGCATGGAAAGTCCCCATAGGGGCGGGGTCATGGAAAGTCCCCATGACCCGGATGTAGCATGGAAAGTCCCCATGGAAAGTCCCCATGGAAAGTCCCCATGTTGCGTGCGAACATTGGGACTTTCCACATTGGGACTTTCCACATTGGGACTTTCCACATTGGGACTTTCCACATGGAAAGTCCCCATGACCCGGATGTAGCATGTTGCGTGCGAACATAGAACGAGGCACATCCCCGGAAGTGACCATAGGGGCGGGGTCATTGGGACTTTCCACATCCCCGGAAGTGACCATTGGGACTTTCCACATGTTGCGTGCGAACATGACCCGGATGTAGCATGGAAAGTCCCCATAGAACGAGGCACATTGGGACTTTCCACATTGGGACTTTCCACATGGAAAGTCCCCATGTTGCGTGCGAACAT
28G6	CATTGGGACTTTCCACATTGGGACTTTCCACATGTTGCGTGCGAACATTGGGACTTTCCACATGGAAAGTCCCCATTGGGACTTTCCACATGGAAAGTCCCCATGGAAAGTCCCCATGACCCGGATGTAGCATTGGGACTTTCCACATCCCCGGAAGTGACCATGTTGCGTGCGAACATGGAAAGTCCCCATTGGGACTTTCCACATGACCCGGATGTAGCATGACCCGGATGTAGCATGACCCGGATGTAGCATGGAAAGTCCCCATAGGGGCGGGGTCATGGAAAGTCCCCATGTTGCGTGCGAACATTGGGACTTTCCACATGTTGCGTGCGAACATCCCCGGAAGTGACCATGGAAAGTCCCCATGGAAAGTCCCCATTGGGACTTTCCACATAGGGGCGGGGTCAT
30D8	CATGGAAAGTCCCCATGTTGCGTGCGAACATTGGGACTTTCCACATAGAACGAGGCACATTGGGACTTTCCACATGACCCGGATGTAGCATGACCCGGATGTAGCATCCCCGGAAGTGACCATTGGGACTTTCCACATGGAAAGTCCCCATGGAAAGTCCCCATAGAACGAGGCACATGACCCGGATGTAGCATTGGGACTTTCCACATGGAAAGTCCCCATTGGGACTTTCCACATAGGGGCGGGGTCATGGAAAGTCCCCATTGGGACTTTCCACATGGAAAGTCCCCATGTTGCGTGCGAACATAGGGGCGGGGTCATGACCCGGATGTAGCATTGGGACTTTCCACATGTTGCGTGCGAACATGGAAAGTCCCCATGGAAAGTCCCCATTGGGACTTTCCACATCCCCGGAAGTGACCATGTTGCGTGCGAACAT
28B7	CATGACCCGGATGTAGCATGGAAAGTCCCCATTGGGACTTTCCACATGACCCGGATGTAGCATGGAAAGTCCCCATTGGGACTTTCCACATTGGGACTTTCCACATGTTGCGTGCGAACATGTTGCGTGCGAACATTGGGACTTTCCACATGTTGCGTGCGAACATCCCCGGAAGTGACCATTGGGACTTTCCACATTGGGACTTTCCACATTGGGACTTTCCACATGGAAAGTCCCCATGGAAAGTCCCCATAGGGGCGGGGTCATTGGGACTTTCCACATCCCCGGAAGTGACCATAGGGGCGGGGTCATGACCCGGATGTAGCATGGAAAGTCCCCATGGAAAGTCCCCATGTTGCGTGCGAACATGGAAAGTCCCCATGGAAAGTCCCCATGACCCGGATGTAGCAT
28D7	CATGGAAAGTCCCCATAGGGGCGGGGTCATGGAAAGTCCCCATGGAAAGTCCCCATGACCCGGATGTAGCATGGAAAGTCCCCATGGAAAGTCCCCATGTTGCGTGCGAACATGGAAAGTCCCCATGACCCGGATGTAGCATGACCCGGATGTAGCATTGGGACTTTCCACATGGAAAGTCCCCATTGGGACTTTCCACATTGGGACTTTCCACATGGAAAGTCCCCATTGGGACTTTCCACATGTTGCGTGCGAACATTGGGACTTTCCACATTGGGACTTTCCACATCCCCGGAAGTGACCATAGGGGCGGGGTCATGTTGCGTGCGAACATGACCCGGATGTAGCATCCCCGGAAGTGACCATTGGGACTTTCCACATTGGGACTTTCCACATGTTGCGTGCGAACAT
28C7	CATGACCCGGATGTAGCATGGAAAGTCCCCATTGGGACTTTCCACATGACCCGGATGTAGCATGGAAAGTCCCCATTGGGACTTTCCACATTGGGACTTTCCACATGTTGCGTGCGAACATGTTGCGTGCGAACATTGGGACTTTCCACATGTTGCGTGCGAACATCCCCGGAAGTGACCATTGGGACTTTCCACATTGGGACTTTCCACATTGGGACTTTCCACATGGAAAGTCCCCATGGAAAGTCCCCATAGGGGCGGGGTCATTGGGACTTTCCACATCCCCGGAAGTGACCATAGGGGCGGGGTCATGACCCGGATGTAGCATGGAAAGTCCCCATGGAAAGTCCCCATGTTGCGTGCGAACATGGAAAGTCCCCATGGAAAGTCCCCATGACCCGGATGTAGCAT
28C8	CATAGGGGCGGGGTCATTGGGACTTTCCACATGTTGCGTGCGAACATTGGGACTTTCCACATGACCCGGATGTAGCATCCCCGGAAGTGACCATGGAAAGTCCCCATGGAAAGTCCCCATTGGGACTTTCCACATGACCCGGATGTAGCATGGAAAGTCCCCATTGGGACTTTCCACATGGAAAGTCCCCATGTTGCGTGCGAACATTGGGACTTTCCACATAGGGGCGGGGTCATTGGGACTTTCCACATTGGGACTTTCCACATGGAAAGTCCCCATGTTGCGTGCGAACATTGGGACTTTCCACATCCCCGGAAGTGACCATGGAAAGTCCCCATGACCCGGATGTAGCATGTTGCGTGCGAACATGGAAAGTCCCCATGGAAAGTCCCCATGACCCGGATGTAGCAT

30B9	CATGGAAAGTCCCCATAGGGGCGGGGTCATGTTGCGTGCGAACATTGGG<br>ACTTTCCACATGGAAAGTCCCCATTGGGACTTTCCACATCCCCGGAAGT<br>GACCATGTTGCGTGCGAACATGTTGCGTGCGAACATGACCCGGATGTAG<br>CATGGAAAGTCCCCATGACCCGGATGTAGCATGACCCGGATGTAGCATT<br>GGGACTTTCCACATTGGGACTTTCCACATGACCCGGATGTAGCATTGGG<br>ACTTTCCACATGTTGCGTGCGAACATGGAAAGTCCCCATGGAAAGTCCC<br>CATTGGGACTTTCCACATAGGGGCGGGGTCATGGAAAGTCCCCATAGAA<br>CGAGGCACATGGAAAGTCCCCATCCCCGGAAGTGACCA7TTGGGACTTT<br>CCACATAGAACGAGGCACATGGAAAGTCCCCATTGGGACTTTCCACAT

Poly (A) tail	sequence
bGH poly(A)	CTGTGCCTTCTAGTTGCCAGCCATCTGTTGTTTGCCCCTCCCCCGTGCCTTCCTTGACCCT<br>GGAAGGTGCCACTCCCACTGTCCTTTCCTAATAAAATGAGGAAATTGCATCGCATTGTCTGA<br>GTAGGTGTCATTCTATTCTGGGGGGTGGGGTGGGGCAGGACAGCAAGGGGGAGGATTGG<br>GAAGACAATAGCAGGCATGCTGGGGATGCGGTGGGCTCTATGG

Lox sites	sequence
LoxP	ATAACTTCGTATAGCATACATTATACGAAGTTAT
Lox2272	ATAACTTCGTATAGGATACTTTATACGAAGTTAT

Primers	sequence	purpose
SSI5_fwd	ACACAGGAGATCTACGCCTCT	5' junction PCR to verify correct integration of mCherry gene
mCherry_rev	TTGGTCACCTTCAGCTTGGC	
Puro_fwd	AGCAACAGATGGAAGGCCTCCT	3' junction PCR to verify correct integration of mCherry gene
SSI5-rev	AGGAGGCAGAGTCAGGTGAAT	
SSI5_out_fwd	TGGACTGCCCTGGAACTCTCTCCATA	Insert PCR to verify correct integration of either msAb or bsAb gene cassettes.
SSI5_out_rev	CTGCCTCATGCACTACATGCATGCGA	

Gene	Forward Primer	Reverse Primer	TaqMan Probe	Dye	Primer Efficiency
mCherry	GCCACTACGACGCTGAGGT	GGTGTAGTCCTCGTTGTGGG	CCAAGAAGCCCGTGC	FAM-MGB	100.2%
msAb_LC	GTGTGCCTGCTGAACAACTTC	ACGCTCTCTTGGCTGTTGC	TTCCACTGCACCTTG	FAM-MGB	107.2%
msAb_HC	ACCTGCGTGGTGGTGGAT	ACTCTTTGCCGTTCAGCCA	CGTACCAATTGAAC	FAM-MGB	102.7%
bsAb_LC1	ACAAAGGTGGAAATCAAAGGC	ATCTGACAAGACAGGGTAGCGT	CCCCTAGATTTCTGACC	FAM-MGB	101.9%
bsAb_HC1	AAGAGCAGGGCAGATTCCA	CGTTTCTCAGGGTCAGAGTGTA	ACGACTCCACCACACT	FAM-MGB	97.2%
bsAb_LC2	CTTCCGTGTTCATCTTCCCA	ATTGTCCACCTTCCACTGCA	CTTCTGTCGTGTGCCT	FAM-MGB	93.4%
bsAb_HC2	CGAGGCGTGATGAAGGACTA	AGAGGTGGATTTAGAGGAAGGAG	TCACCGTGTCCTCT	FAM-MGB	100.6%
COSMC (internal control gene in ddPCR)	ACCCGAACCAGGTAGTAGAA	ACATGTCCAAAGGCCCTAAG	AGTGACAGCCATATTGGAACAGCATCC	VIC-BHQ1	99.1%
Fkbp1a (internal control gene in qPCR)	CTCTCGGGACAGAAACAAGC	GACCTACACTCATCTGGGCTAC	ATGCTAGGCAAGCAGGAGGTGATC	VIC-MGB	100.9%

Supplemental References:

[1] Johari, Y. B., Brown, A. J., Alves, C. S., Zhou, Y., Wright, C. M., Estes, S. D., Kshirsagar, R., & James, D. C. (2019). CHO genome mining for synthetic promoter design. *Journal of biotechnology*, *294*, 1–13.

[2] Robert Gay, Robert Kallmeier, Alison Norman, Stephan Kalwy (2008). Mammalian expression vector comprising the mCMV promoter and first intron of hCMV major immediate early gene (European Patent No. EP-1874929-A2).

[3] Brown, A. J., Gibson, S. J., Hatton, D., & James, D. C. (2017). In silico design of context-responsive mammalian promoters with user-defined functionality. *Nucleic acids research*, *45*(18), 10906–10919.
