# Supplementary material for: Synthetic promoters developed through a site-specific integration system enhance antibody expression in fed-batch culture
Source: Eng Life Sci. 2026 Jul 24;26(7):e005. doi: 10.48130/els-0026-0004 (PMC13599642; doi:10.48130/els-0026-0004)
Supplement: Supplementary file 1 [file els-2026-26-7-0004-S1.zip › 10.48130_els-0026-0004-Suppl-TableS5.pdf]

Table S5: Reporter expression was compared between different synthetic promoters using Mann-Whitney U test. P-values were calculated (corrected for multiple testing using Benjamini-Hochberg FDR).

	28F6	28G6	28B7	28C7	28D7	28-E7	28F7	28G7	28B8	28C8	30D8	30-E8	30F8	30G8	30B9
28F6		0.533	0.071	0.057	0.000	0.000	0.000	0.000	0.194	0.006	0.158	0.319	0.252	0.000	0.030
28G6			0.170	0.158	0.000	0.000	0.000	0.000	0.453	0.008	0.344	0.609	0.451	0.000	0.053
28B7				0.667	0.015	0.000	0.003	0.006	0.392	0.229	0.848	0.479	0.420	0.048	0.763
28C7					0.242	0.006	0.063	0.130	0.308	0.620	0.603	0.423	0.381	0.420	0.835
28D7						0.000	0.170	0.507	0.000	0.420	0.015	0.022	0.000	0.451	0.008
28-E7							0.506	0.032	0.000	0.000	0.000	0.000	0.000	0.000	0.000
28F7								0.423	0.000	0.060	0.003	0.003	0.000	0.039	0.000
28G7									0.000	0.180	0.006	0.006	0.000	0.171	0.003
28B8										0.013	0.620	0.967	0.914	0.000	0.180
28C8											0.160	0.130	0.034	0.736	0.270
30D8												0.809	0.740	0.053	0.623
30-E8													0.967	0.043	0.392
30F8														0.000	0.259
30G8															0.030
30B9															
