# Supplementary material for: Synthetic promoters developed through a site-specific integration system enhance antibody expression in fed-batch culture
Source: Eng Life Sci. 2026 Jul 24;26(7):e005. doi: 10.48130/els-0026-0004 (PMC13599642; doi:10.48130/els-0026-0004)
Supplement: Supplementary file 1 [file els-2026-26-7-0004-S1.zip › 10.48130_els-0026-0004-Suppl-TableS6.pdf]

Table S6: Median of reporter expression level driven by different promoters were listed. Reporter expression level was compared between synthetic promoters and CMV using Mann-Whitney U test. P-values were corrected for multiple testing using Benjamini-Hochberg FDR.

Promoter	Median	Compare with CMV	p-value
28F6	409.571	>	0.006
28G6	379.624	>	0.005
28B7	311.273	≠	0.533
28C7	281.272	≠	0.994
28D7	237.564	<	0.000
28-E7	182.541	<	0.000
28F7	206.831	<	0.000
28G7	221.794	<	0.000
28B8	361.261	>	0.011
28C8	254.065	≠	0.273
30D8	328.793	≠	0.403
30-E8	319.892	≠	0.282
30F8	326.183	>	0.023
30G8	255.219	<	0.003
30B9	330.033	≠	0.619
CMV	306.039	N/A	N/A
