# Supplementary material for: Synthetic promoters developed through a site-specific integration system enhance antibody expression in fed-batch culture
Source: Eng Life Sci. 2026 Jul 24;26(7):e005. doi: 10.48130/els-0026-0004 (PMC13599642; doi:10.48130/els-0026-0004)
Supplement: Supplementary file 1 [file els-2026-26-7-0004-S1.zip › 10.48130_els-0026-0004-Suppl-TableS7.pdf]

Table S7: Copy number of mCherry in different monoclonal cells. Ratio between 0.5 to 1.5 is considered as single copy (Gene copy number is defined as an integer. We defined a ratio between 0.5 to 1.5 as single copy; a ratio between 1.5 to 2.5 as two copies; and so on. This threshold setting method can evenly divide the space between adjacent copy number.).

Clone ID	mcherry (FAM) 1 μL DNA final copies	COSMC (VIC) 1 μL DNA final copies	Target-reference ratio (mCherry copy #)
28F6_1	2648	2468	1.07
28F6_2	10720	10460	1.02
28F6_3	9420	8000	1.18
28E7_1	8300	8220	1.01
28E7_2	14700	13480	1.09
28E7_3	12660	12360	1.02
28F7_1	3608	3576	1.01
28F7_2	11020	10040	1.10
28F7_3	11380	10840	1.05
28G7_1	10080	9240	1.09
28G7_2	9000	9460	0.95
28G7_3	14060	12660	1.11
28B8_1	11500	11180	1.03
28B8_2	17660	16100	1.10
28B8_3	9500	9720	0.98
30E8_1	4864	4048	1.20
30E8_2	14900	14940	1.00
30E8_3	9380	16680	0.56
30F8_1	6600	6460	1.02
30F8_2	8560	7580	1.13
30F8_3	7960	7520	1.06
30G8_1	2324	2044	1.14
30G8_2	3804	3580	1.06
30G8_3	1804	1736	1.04
