# Supplementary material for: Synthetic promoters developed through a site-specific integration system enhance antibody expression in fed-batch culture
Source: Eng Life Sci. 2026 Jul 24;26(7):e005. doi: 10.48130/els-0026-0004 (PMC13599642; doi:10.48130/els-0026-0004)
Supplement: Supplementary file 1 [file els-2026-26-7-0004-S1.zip › 10.48130_els-0026-0004-Suppl-TableS8.pdf]

Table S8: Ratio of MFI from W9 to W0

W9/W0	Monoclone-1	Monoclone-2	Monoclone-3
28F6	0.747	1.238	1.280
28-E7	1.243	1.288	1.074
28F7	0.915	1.455	1.257
28G7	1.426	1.364	1.350
28B8	1.402	1.360	1.483
30-E8	1.602	0.941	0.957
30F8	0.981	0.985	1.034
30G8	1.105	1.128	1.023
