# Supplementary material for: Synthetic promoters developed through a site-specific integration system enhance antibody expression in fed-batch culture
Source: Eng Life Sci. 2026 Jul 24;26(7):e005. doi: 10.48130/els-0026-0004 (PMC13599642; doi:10.48130/els-0026-0004)
Supplement: Supplementary file 1 [file els-2026-26-7-0004-S1.zip › 10.48130_els-0026-0004-Suppl-TableS9.pdf]

Table S9: Estimate of correct integration ratio of msAb genes cassette before PCR test.

Promoter type	# of monoclonal cells sorted by FACS	# of survived monoclonal cells	# of cells with FITC- & titer > 3 μg/mL	% of correct integration
28-F6	192	72	34	47
28-E7	192	50	32	64
28-F7	192	96	52	54
28-G7	192	67	26	39
28-B8	192	72	27	38
30-E8	192	63	24	38
30-F8	192	67	29	43
30-G8	192	66	31	47
CMV	192	64	9	14
